# Versatile tissue‐injectable hydrogels capable of the extended hydrolytic release of bioactive protein therapeutics

**DOI:** 10.1002/btm2.10668

**Published:** 2024-04-15

**Authors:** Eric S. Nealy, Steven J. Reed, Steven M. Adelmund, Barry A. Badeau, Jared A. Shadish, Emily J. Girard, Kenneth Brasel, Fiona J. Pakiam, Andrew J. Mhyre, Jason P. Price, Surojit Sarkar, Vandana Kalia, Cole A. DeForest, James M. Olson

**Affiliations:** ^1^ Seattle Children's Research Institute Seattle Washington USA; ^2^ Fred Hutch Cancer Center Seattle Washington USA; ^3^ Department of Chemical Engineering University of Washington Seattle Washington USA; ^4^ Department of Pathology University of Washington Seattle Washington USA; ^5^ Department of Pediatrics University of Washington Seattle Washington USA; ^6^ Department of Bioengineering University of Washington Seattle Washington USA; ^7^ Department of Biochemistry University of Washington Seattle Washington USA; ^8^ Department of Chemistry University of Washington Seattle Washington USA; ^9^ Institute for Stem Cell and Regenerative Medicine, University of Washington Seattle Washington USA; ^10^ Institute for Protein Design, University of Washington Seattle Washington USA; ^11^ Department of Pharmacology University of Washington Seattle Washington USA

**Keywords:** drug delivery, extended release, hydrogels, immuno‐oncology, loco‐regional, proteins, sortase

## Abstract

Hydrogels are extensively employed in healthcare due to their adaptable structures, high water content, and biocompatibility, with FDA‐approved applications ranging from spinal cord regeneration to local therapeutic delivery. However, clinical hydrogels encounter challenges related to inconsistent therapeutic exposure, unmodifiable release windows, and difficulties in subsurface polymer insertion. Addressing these issues, we engineered injectable, biocompatible hydrogels as a local therapeutic depot, utilizing poly(ethylene glycol) (PEG)‐based hydrogels functionalized with bioorthogonal SPAAC handles for network polymerization and functionalization. Our hydrogel solutions polymerize in situ in a temperature‐sensitive manner, persist in tissue, and facilitate the delivery of bioactive therapeutics in subsurface locations. Demonstrating the efficacy of our approach, recombinant anti‐CD47 monoclonal antibodies, when incorporated into subsurface‐injected hydrogel solutions, exhibited cytotoxic activity against infiltrative high‐grade glioma xenografts in the rodent brain. To enhance the gel's versatility, recombinant protein cargos can undergo site‐specific modification with hydrolysable “azidoester” adapters, allowing for user‐defined release profiles from the hydrogel. Hydrogel‐generated gradients of murine CXCL10, linked to intratumorally injected hydrogel solutions via azidoester linkers, resulted in significant recruitment of CD8^+^ T‐cells and the attenuation of tumor growth in a “cold” syngeneic melanoma model. This study highlights a highly customizable, hydrogel‐based delivery system for local protein therapeutic administration to meet diverse clinical needs.

**Translational Impact Statement**

We have developed injectable hydrogels for local, controlled release of protein therapeutics into tissue, particularly beneficial for potent immunotherapies with severe systemic toxicities. The hydrogel, featuring slowly hydrolyzing linkers, prolongs the therapeutic effect, minimizing the requirement for repeated surgeries. Designed for subsurface injection, our hydrogel provides a customizable range of release profiles, lasting from days to over a month as needed, and exhibits compatibility with a wide array of therapeutically relevant proteins, granting clinicians versatile solutions for diverse circumstances.

## 1 INTRODUCTION

Delivery of therapeutic agents directly into tissue or body compartments allows clinicians to achieve higher local drug concentrations than could be achieved by systemic administration, which is hindered by dose‐limiting toxicities (DLTs).^1^, ^2^, ^3^, ^4^, ^5^ Hematologic, hepatic, and neurologic effects are common examples of DLTs that may prevent achieving effective drug concentrations following systemic delivery.^6^, ^7^ This has led to the development of novel local or regional delivery strategies aimed at minimizing DLTs and improving therapeutic efficacy. Loco‐regional delivery may be particularly suited for protein‐based therapeutics, such as monoclonal antibodies and cytokines. Although they have high potency and target specificity, their systemic administration can cause damage to healthy tissues, particularly due to on‐target/off‐cancer, or similar, toxicities.^8^, ^9^, ^10^, ^11^, ^12^, ^13^, ^14^, ^15^, ^16^, ^17^, ^18^, ^19^

Hydrogels, a class of biomaterials comprised of water‐swollen polymer networks, are commonly used to deliver therapeutics locally to tissue surrounding their implant site.^20^, ^21^, ^22^ These materials can swell with fluid from their environment, enabling the exchange of nutrients and molecules between tissue and the gel.^23^, ^24^, ^25^, ^26^, ^27^, ^28^, ^29^, ^30^, ^31^, ^32^, ^33^, ^34^, ^35^, ^36^, ^37^ Additionally, hydrogels offer unique structural flexibility, with mechanical properties and environmental responsiveness that can be adjusted to suit various biological settings. Our groups are particularly interested in the potential of these implantable materials for deploying immunotherapy, as they have demonstrated effectiveness in delivering immunostimulatory proteins across various delivery modalities and in numerous preclinical cancer models.^38^, ^39^, ^40^, ^41^, ^42^, ^43^, ^44^, ^45^, ^46^, ^47^ Despite their clinical utility, there remain drawbacks to therapeutic delivery via hydrogels. Many hydrogel‐housed therapeutics rely on structural degradation for extended release into tissue, leading to non‐customizable release rates, short‐lived therapeutic exposure, and/or uneven therapeutic secretion at the degrading implant‐tissue interface.^4^, ^5^, ^25^, ^48^, ^49^, ^50^ Moreover, several studies deploy their gels into superficial flank tumors or into large cavities within tissue, which may not always be feasible in human patients.^38^, ^39^, ^40^, ^41^, ^42^, ^43^, ^44^, ^45^, ^46^, ^47^ Addressing these concerns may be crucial in some clinical contexts, particularly when administering therapeutic combinations with differing effective concentrations or in locations where extensive surgeries are not practical.

Our team has developed a highly customizable hydrogel platform to overcome several of these limitations. This system utilizes poly(ethylene glycol) (PEG)‐based hydrogels polymerized with bioorthogonal SPAAC click chemistry, allowing for subsurface tissue injectability, user‐defined payload release rate capabilities, and broad compatibility with therapeutically relevant proteins without compromising their bioactivity.^51^, ^52^, ^53^ We genetically encoded recombinant immunostimulatory proteins, including an immune checkpoint blocking antibody and classical chemokines, with C‐terminal sortase‐recognition sequences (i.e., LPXTG, where X is any amino acid). These modifications enable their site‐specific modification with azide‐functionalized peptide adapters (PolyG‐azidoesters) through chemoenzymatic transpeptidation, conferring SPAAC hydrogel compatibility and, if desired, user‐defined hydrolysis rates based on azidoester hydrophobicity.^30^, ^54^, ^55^ Recombinant anti‐CD47 monoclonal antibodies, when admixed within subsurface‐injected hydrogel solutions, demonstrated cytotoxic activity against an infiltrative high‐grade glioma xenograft. Hydrogel‐generated gradients of murine CXCL10, conjugated to intratumorally injected hydrogel solutions via PolyG‐azidoesters, elicited the recruitment of CD8^+^ T‐cells in a “cold” syngeneic melanoma model. Our results suggest that our system simplifies the incorporation of a variety of therapeutic delivery attributes, ranging from injectability into subsurface tissues to user‐defined release rate customization and protein compatibility, into one hydrogel that functions in a “fire‐and‐forget” manner.

## 2 RESULTS

### 2.1 Characterization and the temperature‐sensitive polymerization of PEG‐tetraBCN hydrogels in the subsurface tissue

**FIGURE 1:**
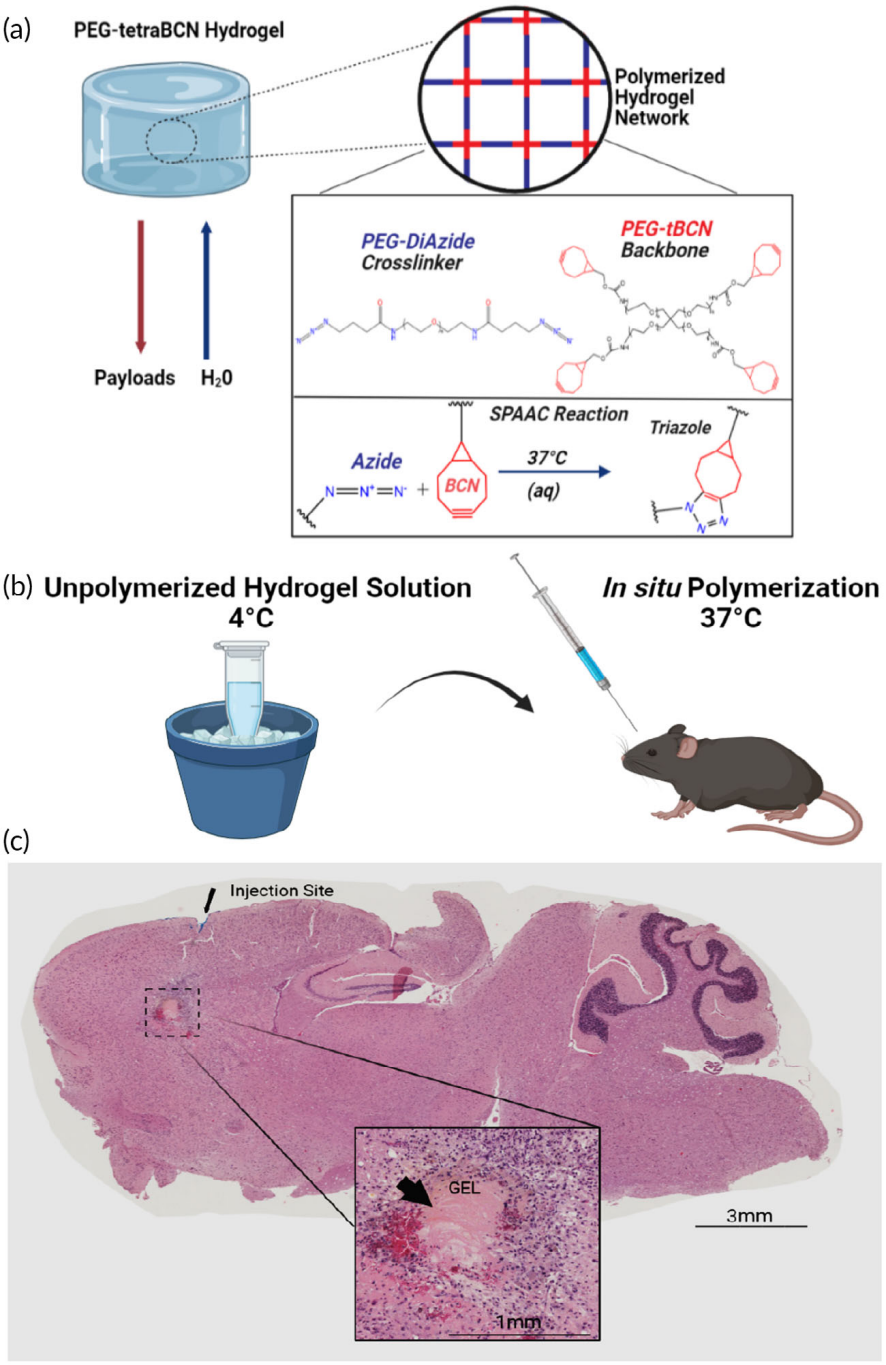
Characteristics of a bioorthogonal hydrogel with customizable payload release rates (A) Illustration depicting the components of a PEG‐tetraBCN hydrogel and its individual reactants.^29^, ^32^, ^34^ The 4‐armed PEG‐tetraBCN backbone is labeled red and the linear PEG‐diazide crosslinker is in blue. The temperature sensitive SPAAC reaction between a BCN group and an azide result in a covalent triazole adduct, polymerizing the gel.^51^, ^58^, ^59^ (B) Mixing the individual gel components on ice slows the SPAAC reaction, allowing additional time for aspirating the gel mixture into a syringe and prompt injection into an animal. The optimal polymerization temperature is reached upon exposure to physiological body temperature. (C) Chilled PEG‐tetraBCN hydrogel solutions were administered into the frontal cortex of living mice using a silanized Hamilton syringe. After 7 days, brains were harvested, sectioned, and later examined via H&E. The intact gel was found within the injection tract. Larger image taken at 10× magnification.

Our study aims to showcase the utilization of four‐arm PEG‐tetraBCN (PEG‐tBCN) hydrogels (*M*_n_ ≈ 20,000 Da) as an exceptionally customizable and bioorthogonal platform for protein therapeutic delivery in a variety of clinically relevant scenarios.^29^, ^32^, ^56^, ^57^ The hydrogel network is polymerized via strain‐promoted azide‐alkyne cycloaddition (SPAAC), employing a PEG‐diazide crosslinker (*M*_n_ ≈ 3400 Da) to covalently link together the BCN groups that are functionalized to the PEG backbone without requiring a catalyst (Figure 1a). SPAAC, a catalyst‐free click reaction that proceeds in mild aqueous conditions at 37°C, exhibits high compatibility with various chemical moieties and bioactive molecules.^29^, ^32^, ^51^, ^58^

A key feature of this gel chemistry is its temperature sensitivity. Formulating the hydrogel solution at 4°C slows the SPAAC reaction rate, enabling aspiration and injection into tissue, followed by in vivo polymerization (Figure 1b).^59^ This has translational potential, particularly for areas of the body where there is no cavity present or where the implantation of a 3D solid polymer is impractical. We chose the brain as an illustrative example, recognizing the physical constraints of the cranium and neuronal sensitivity to pressure, highlighting challenges associated with solid object implantation. Our studies showcase the feasibility of injecting complete PEG‐tetraBCN hydrogel solutions into living brain tissue without complex steps. Chilled 6.5% (w/v) PEG‐tetraBCN gel solutions on ice were aspirated into silanized Hamilton syringes and deposited as 3 μL solutions into living mouse brains at depths ranging from 1 to 3 mm (Figures 1c and S1). After injection, the animal's body temperature allows the SPAAC reaction to proceed at its optimal rate, and the newly polymerized gel forms within the needle tract of the injection site. The mice survived for the entire weeklong study without any visible adverse effects from the gel in their brains, owing to inherent biocompatibility of the PEG gel components and SPAAC gelation chemistry.

### 2.2 Macrophage checkpoint blockers diffusing from cortically injected hydrogels exhibit activity against infiltrative high‐grade glioma xenografts

**FIGURE 2:**
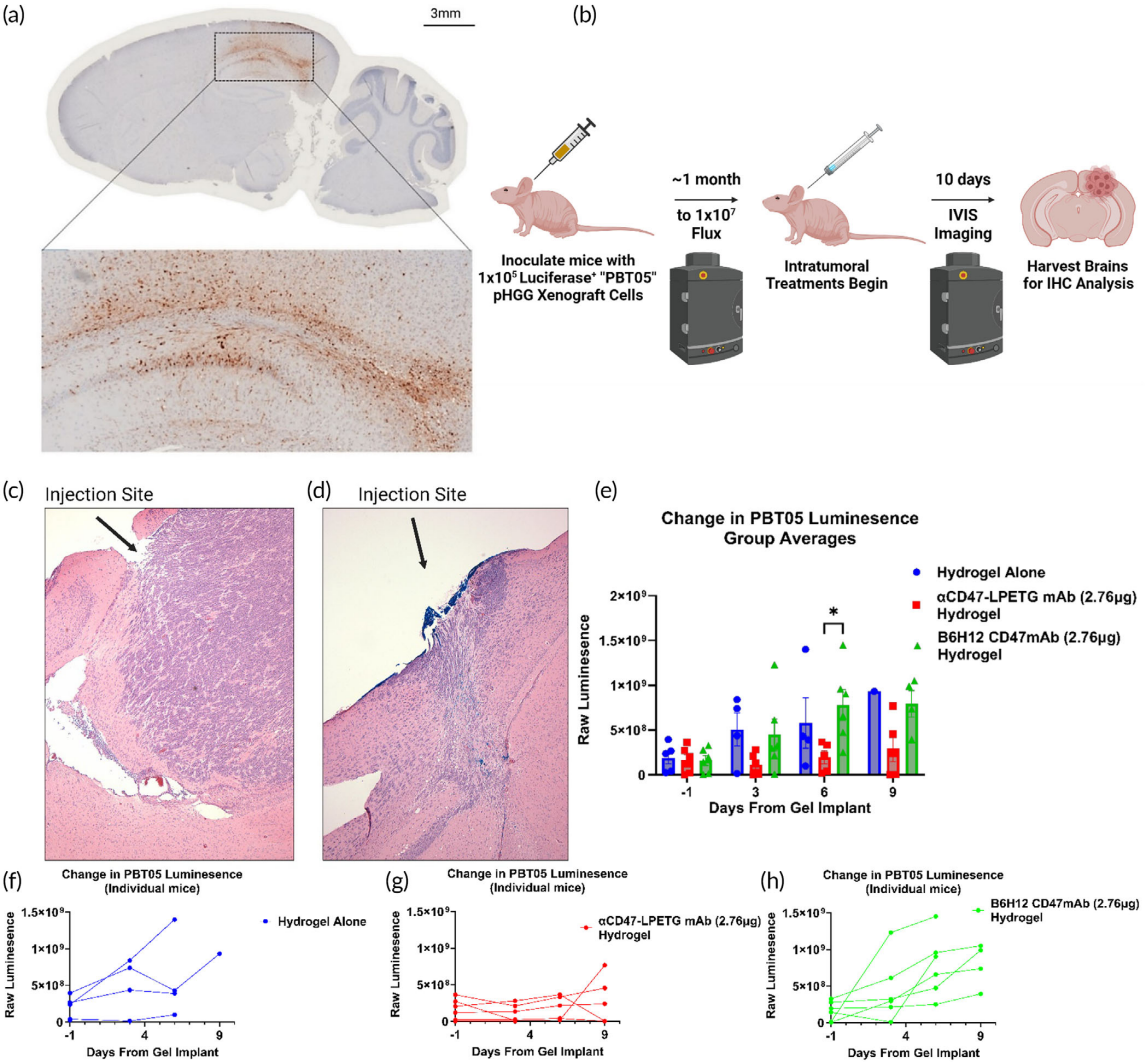
Immune checkpoint blockade antibodies maintain bioactivity after diffusing from the hydrogel (A) mCherry‐DAB staining illustrating the infiltrative nature of a pHGG xenograft tumor, PBT05, in the murine cortex, stained with DAB‐mCherry. (B) Illustration of the hydrogel cortical injection study design, including the timing of Luc^+^ PBT05 pHGG inoculations and hydrogel injection. Treatments: Hydrogel alone control, B6H12 CD47mAb hydrogel, and in‐house recombinant 2.3D11 αCD47‐LPETG mAb hydrogel. (C, D) Representative H&E of murine brains with PBT05 xenografts injected with hydrogel alone (Left) or αCD47‐LPETG admixed within a hydrogel solution (Right). (E) Summarized results of the 10‐day study demonstrating equivalence between in‐house αCD47‐LPETG mAb and the B6H12 commercial alternative. Luminescence values over 10 days for mice bearing PBT05 xenograft tumors. Data are presented as mean ± SEM for *n* = 6 initial replicates. Statistical significance was determined by Mixed Effects two‐way ANOVA followed by Holm–Šídák post‐hoc correction. (F–H) Data from each individual mouse and their treatment groups presented in E. Only one mouse in the hydrogel alone group survived to study endpoint, 4 and 5 mice survived in the B6H12 and αCD47‐LPETG hydrogel conditions, respectively. [(*) *p* < 0.05.]

We replicated the cortical hydrogel injection study, this time utilizing mice burdened with Luciferase^+^ pediatric high‐grade glioma xenografts. These patient‐derived tumors infiltrate the murine brain in a similar fashion to the human brain, exhibiting a diffusely infiltrative migration pattern with minimal localization to superficial locations, as would be observed in flank tumor models (Figure 2a). This high‐grade glioma overexpresses the CD47 ligand on its surface (Figure S2). This is an often‐targeted ligand in immunotherapy, as it is typically overexpressed on tumor cells and causes inhibitory “don't eat me” signaling cascades in phagocytic cells like macrophages.^11^, ^43^, ^60^, ^61^, ^62^, ^63^, ^64^, ^65^ We demonstrate these tumor cells are rapidly phagocytosed in vitro when co‐cultured with murine macrophages treated with commercially available anti‐CD47 monoclonal antibodies (mAbs) (Figure S2). However, as CD47 is also expressed on noncancerous cells, such as platelets and red blood cells, delivering this antibody locally via our gel, rather than systemically, is advantageous to avoid known on‐target‐off‐cancer toxicities.^14^, ^66^, ^67^ We aimed to confirm the biological activity of anti‐CD47mAbs against subsurface xenograft tumors when delivered from PEG‐tBCN hydrogels polymerized within brain tissue. Chilled 6.5% (w/v) hydrogel solutions, admixed with PBS, an in‐house recombinant anti‐CD47mAb 2.3D11 clone (“αCD47mAb‐LPETG”), or a commercially available anti‐CD47mAb B6H12 clone, were injected ~2 mm subsurface into the brain through the same bore hole as the xenograft cell implantation (Figures 2b–d, S3 and S4). Over the span of a week, we observed growth attenuation of the Luciferase^+^ xenografts in both CD47mAb treatments via IVIS imaging, indicating the maintained potency of two distinct clones of the anti‐CD47mAb despite their prior mixing and delivery from an injected hydrogel (Figure 2e–h).

### 2.3 Bestowing SPAAC chemical compatibility to protein payloads via polypeptide adapters

**FIGURE 3:**
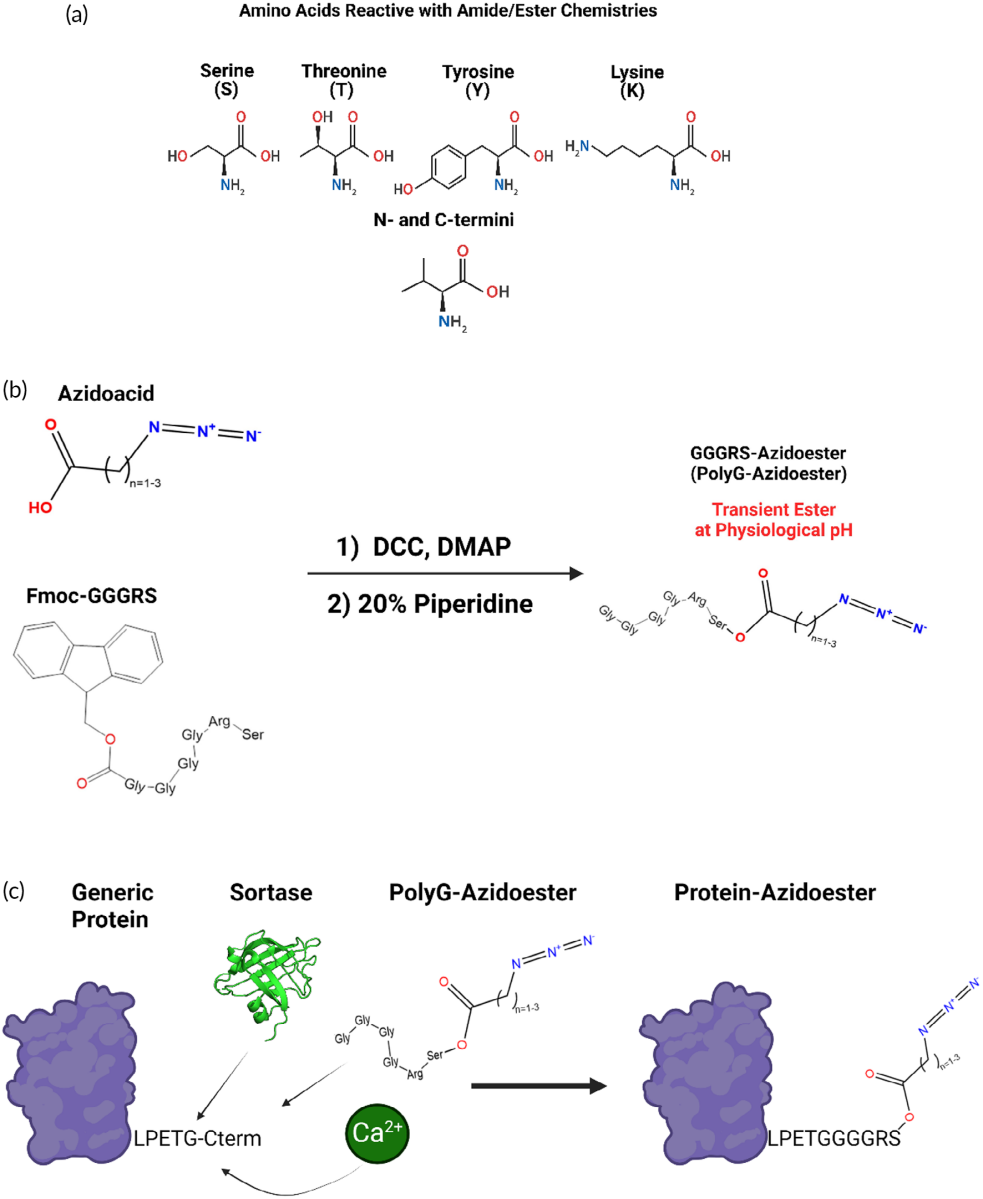
Synthesizing polypeptide adapter molecules to facilitate protein incorporation into PEG‐tBCN Hydrogels (A) Illustration showing possible ester and amide chemical conjugation sites on amino acids and N/C‐termini.^28^, ^30^, ^71^ (B) Polypeptides with specified reactive residues can be chemically modified with azide species to become chemically compatible with SPAAC. We synthesized the Fmoc‐protected GGGGRS polypeptide (PolyG) to enable site specific ester linkage (PolyG‐Azidoester). The N‐terminus can then be deprotected and utilized in sortase tagging “sortagging” to a protein of interest. (C) Schematic demonstrating the sortagging of PolyG‐Azidoester to a generic protein of interest at its C‐terminal LPXTG sortase recognition motif to create a protein capable of binding to a SPAAC hydrogel, like PEG‐tBCN.^72^, ^73^

Linear ω‐terminal azide‐functionalized fatty acids (azido acids) exhibit chemical compatibility with the PEG‐tBCN hydrogel backbone, like the PEG‐diazide crosslinker used in gel polymerization (Figure 1a).^68^, ^69^, ^70^ We utilized these fatty acids in our studies as linkers between a therapeutic of choice and the hydrogel backbone. Commonly used bioconjugation chemistries, like *N*‐hydroxysuccinimide (NHS) ester chemistry or carbodiimide‐mediated coupling, may not always be suitable for coupling azido acids to bioactive proteins, as random attachments can lead to disrupted biological activity (Figure 3a).^28^, ^30^, ^74^ To address this, we synthesized the Fmoc protected “H‐GGGGRS‐NH_2_” polypeptide (“PolyG”), whose sole C‐terminal serine can undergo esterification reactions with an azido acid (Figure 3b, Methods S1–S2, Figure S5).^71^, ^74^, ^75^, ^76^, ^77^, ^78^, ^79^ After Fmoc deprotection and peptide purification, the N‐terminal GGG‐ can be enzymatically coupled to a chosen recombinant protein genetically encoded with a C‐terminal LPXTG motif through sortase‐mediated coupling, commonly known as “sortase tagging” or “sortagging” (Figure 3c).^30^, ^54^, ^55^, ^71^, ^80^, ^81^, ^82^, ^83^ Ultimately, modifying polypeptides containing specifically chosen reactive sites, like H‐GGGGRS‐NH_2_, with an azido acid can enable the rapid generation of adapter molecules that facilitate the linkage of recombinant protein therapeutics to the PEG‐tBCN backbone in a site‐specific manner. This sortase tagging method is amenable to a wide variety of protein types and expression systems, and we next highlight distinct methods of modifying both mammalian and bacteria‐derived proteins: Traditional sortase tagging and Sortase‐Tagged Expressed Protein Ligation (STEPL).^30^, ^54^, ^55^

### 2.4 Generating SPAAC compatible macrophage checkpoint blockers via traditional sortase tagging

**FIGURE 4:**
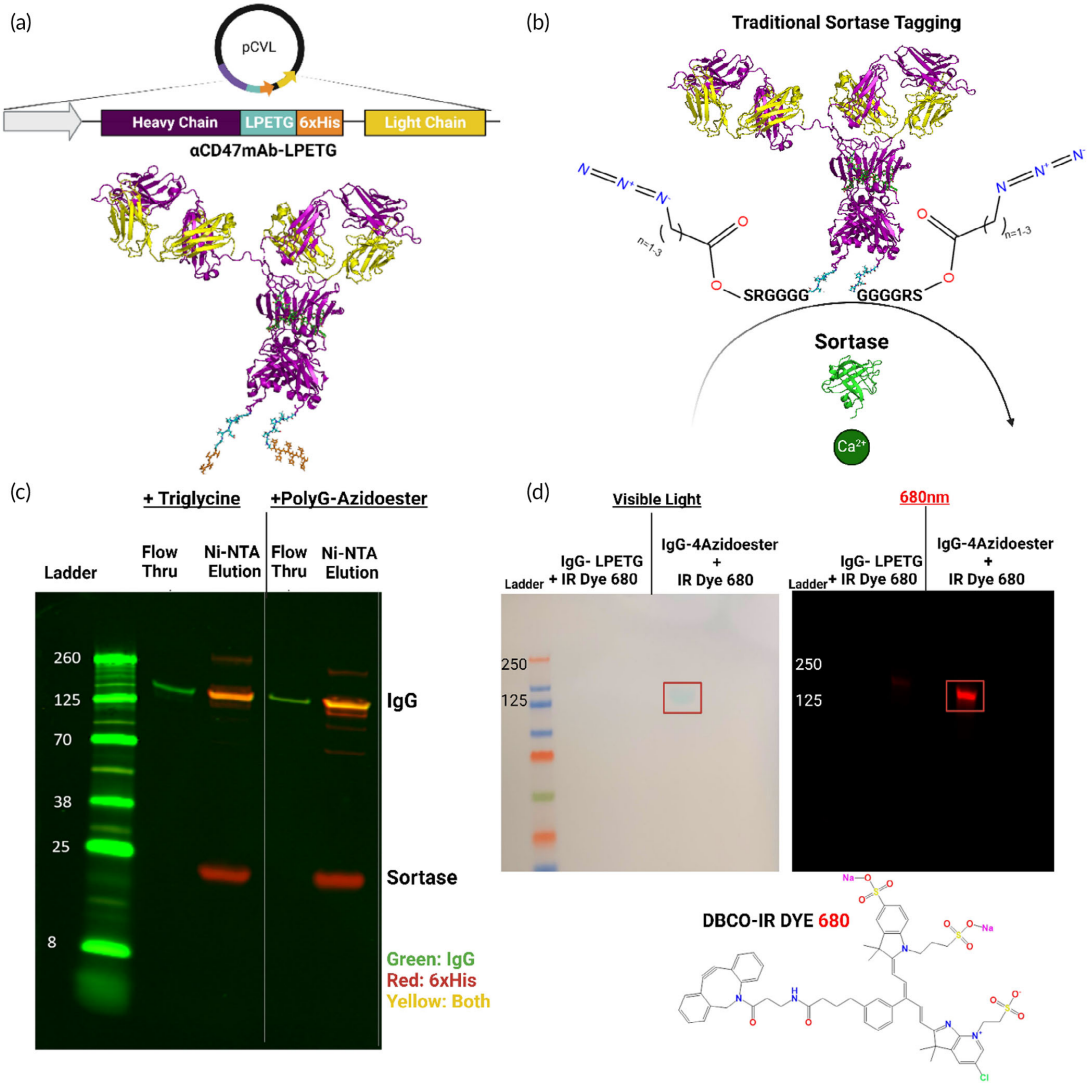
Confirming SPAAC compatibility of bioactive immune checkpoint antibodies **(**A‐B) Illustrations of αCD47‐LPETG expression via the Daedalus mammalian expression platform and its modification with PolyG‐4Azidoesters.^72^, ^73^, ^84^, ^85^, ^86^ “Traditional” sortase tagging was utilized, requiring separate expression of sortase and the protein target of interest prior to tagging with GGG‐ containing species.^54^ (C) This Western blot confirms the removal of both 6xHis tags (red channel) from αCD47‐LPETG (green channel) after sortagging with a triglycine positive control or PolyG‐4Azidoester. Sortagged proteins flow through a Ni‐NTA column, while unmodified or partially modified proteins with 6xHis tags remain attached to the column. (Right) This Western blot compares the parental αCD47‐LPETG with αCD47‐4azidoester after overnight reaction with DBCO‐IRDye 680. An accumulation of dye appears at the MW of an IgG (~150 kDa) in the αCD47‐4Azidoester lane only. Scanning this blot in the 680 (red) channel confirms a signal from the dye at the MW of αCD47‐4azidoester.

The traditional sortase tagging technique, as originally described by Popp *et.al*, relies on the interactions between a N‐terminal GGG‐ containing species, a recombinant protein encoded with a C‐terminal LPXTG motif, and a separately expressed sortase enzyme.^54^, ^55^ This technique is widely applicable to various recombinant proteins, including commonly used immunotherapies with recognized systemic toxicities, such as immune cell‐engaging proteins.^19^ We applied this method to our in‐house recombinant anti‐CD47mAb derived from the 2.3D11 clone, “αCD47‐LPETG”, utilized in Figure 2. This antibody was encoded with LPETG sortase motifs and 6xHis tags on its heavy chain C‐termini (Figures 4a, and S4).^54^, ^80^ The C‐terminal sortase extensions (and any subsequent sortase‐mediated modifications) are distal to the CH_2_ domain that interacts with the FcγR, as well as the antibody's antigen binding domains (Figures 4b and S4). Following the reaction of αCD47‐LPETG with sortase and either a triglycine control or a PolyG‐azidoester, nonreactivity to the 6xHis antibody on a western blot verified the removal of the 6xHis tag, along with its ability to flow through a Ni‐NTA column. Unmodified or partially modified antibodies were recovered with Ni‐NTA elution buffer and maintained reactivity with the 6xHis antibody (Figure 4c). We verified the presence of an azide attached to the antibody in this process and ensured that the 6xHis tag was not simply cleaved off at the LPETG motif without transpeptidation, as sortase is capable of doing under certain circumstances.^87^, ^88^ An overnight click reaction was conducted between both unmodified αCD47‐LPETG and αCD47‐4azidoester with DBCO‐IRDye 680. DBCO, like BCN, is a ring‐strained molecule compatible with SPAAC click chemistry.^89^ Western blot analysis revealed a 680 nm signal, approximately the expected size of an IgG (~150 kDa), exclusively in the lane containing αCD47‐4azidoester (Figure 4d).

### 2.5 Payload release customization from PEG‐tBCN hydrogels via azidoester hydrolysis

**FIGURE 5:**
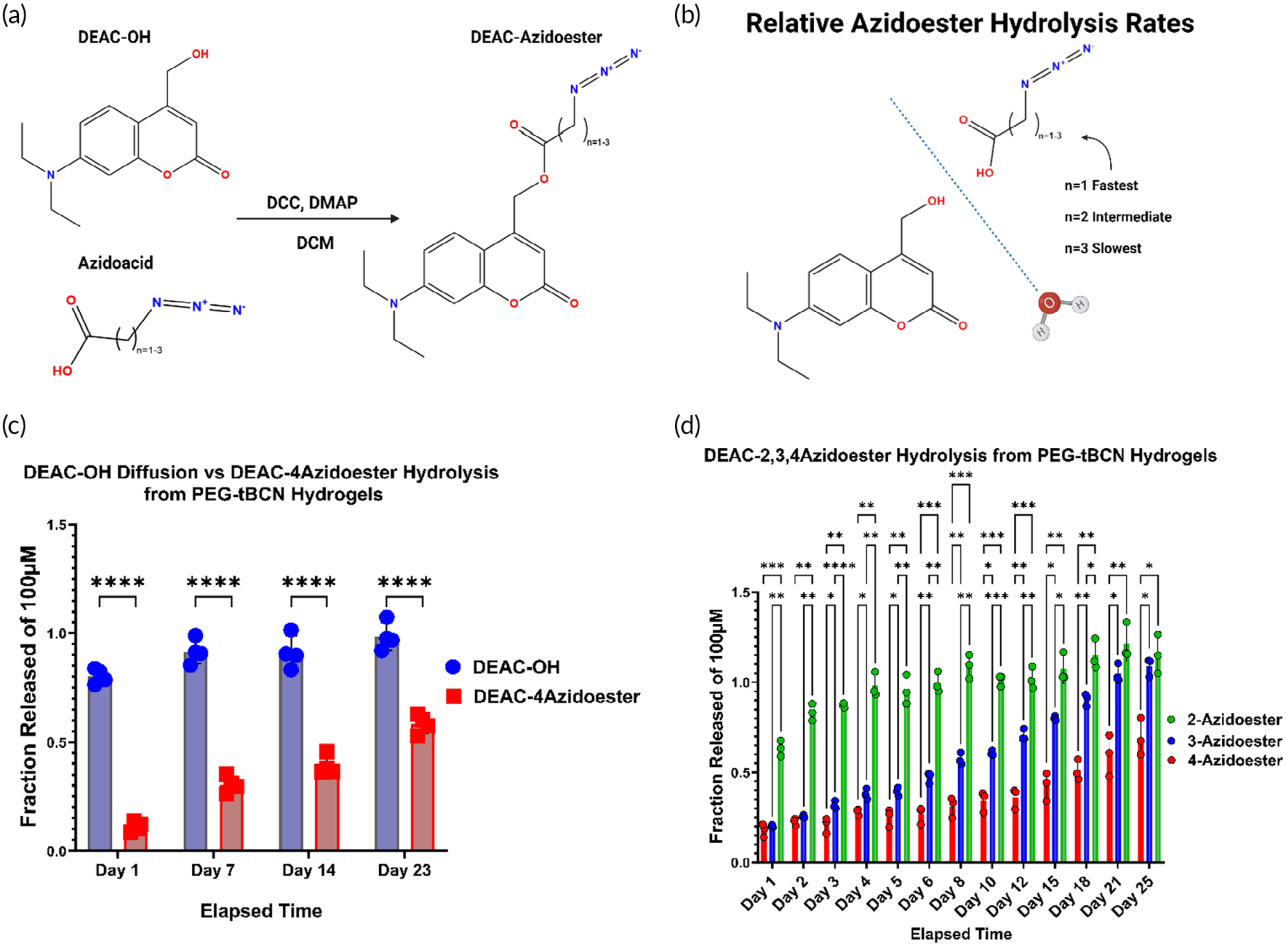
Azidoester hydrolysis allows for customizable payload release rates (A) Steglich esterification converts DEAC‐OH and an azido acid into DEAC‐azidoesters.^71^ (B) Ester bonds linking DEAC to an azidoacid hydrolyze in aqueous environments at rates dependent on acid hydrophobicity.^79^ (C) Comparison of DEAC‐OH diffusion release profile from a hydrogel versus DEAC‐4Azidoester hydrolysis from a PEG‐tBCN hydrogel. Data are presented as mean ± SD for *n* = 4 replicates. Statistical significance was determined by multiple unpaired *t*‐tests followed by Holm–Šídák post‐hoc correction. (D) Simultaneous release assay from multiple gel conditions demonstrates the customizability of the azidoester system, with 2‐Azidoesters completely releasing rapidly and 4‐Azidoesters releasing over multiple weeks.^79^ Data are presented as mean ± SD for *n* = 4 replicates. Statistical significance was determined by two‐way ANOVA followed by followed by Holm–Šídák post‐hoc correction. [(*) *p* < 0.1, (**) *p* < 0.01, (***) *p* < 0.001, (****), and *p* < 0.0001].

Azidoesters, formed by ester bonds between an azido acid and the payload, hydrolyze at rates dependent on fatty acid's hydrophobicity, aligning with trends reported for similar linker chemistries.^79^ Therefore, the release profile of an azidoester‐linked therapeutic payload from the PEG‐tBCN hydrogel can be pre‐determined. Linear, ω‐terminal azido acids increase in hydrophobicity based on the length of their acyl chain, allowing for straightforward choices when customizing payload hydrolysis rates.^90^, ^91^ We initially demonstrate this customizability in vitro with the fluorescent small molecule DEAC‐OH (MW = 247.29 Da) serving as a model payload (Figure 5a, Method S3). We performed esterification reactions between DEAC‐OH and linear, ω‐terminal azido acids of varying lengths to yield what we refer to as “DEAC‐azidoesters” (Figure 5b).^71^, ^78^ We analyzed the release of the payload over 4 weeks from gels immersed in phosphate‐buffered saline (PBS) release media at 37°C. Initial spectroscopy of the supernatant revealed approximately 80% of unlinked DEAC‐OH diffused from the gel in less than 24 h. However, by day 23 of the study, ~60% of the DEAC‐4azidoester payload, originally conjugated to the hydrogel backbone, was released (Figure 5c). Payload release rates varied based on the length of the esterified azidoacid, providing user‐defined control over hydrolysis from the gel backbone. We esterified DEAC‐OH with either 2‐azidoacetic, 3‐azidobutanic, or 4‐azidopropanic acids and we quantified their respective release from PEG‐tBCN hydrogels over 4 weeks into PBS. Complete DEAC release occurred within 5 days using 2‐azidoacetic acid, 21 days for the 3‐azidobutanic acid, and only ~69% released using the 4‐azidopropanic acid by day 25 (Figure 5d).

### 2.6 Achieving prolonged release of chemokines from PEG‐tBCN hydrogels through STEPL of polypeptide adapters

**FIGURE 6:**
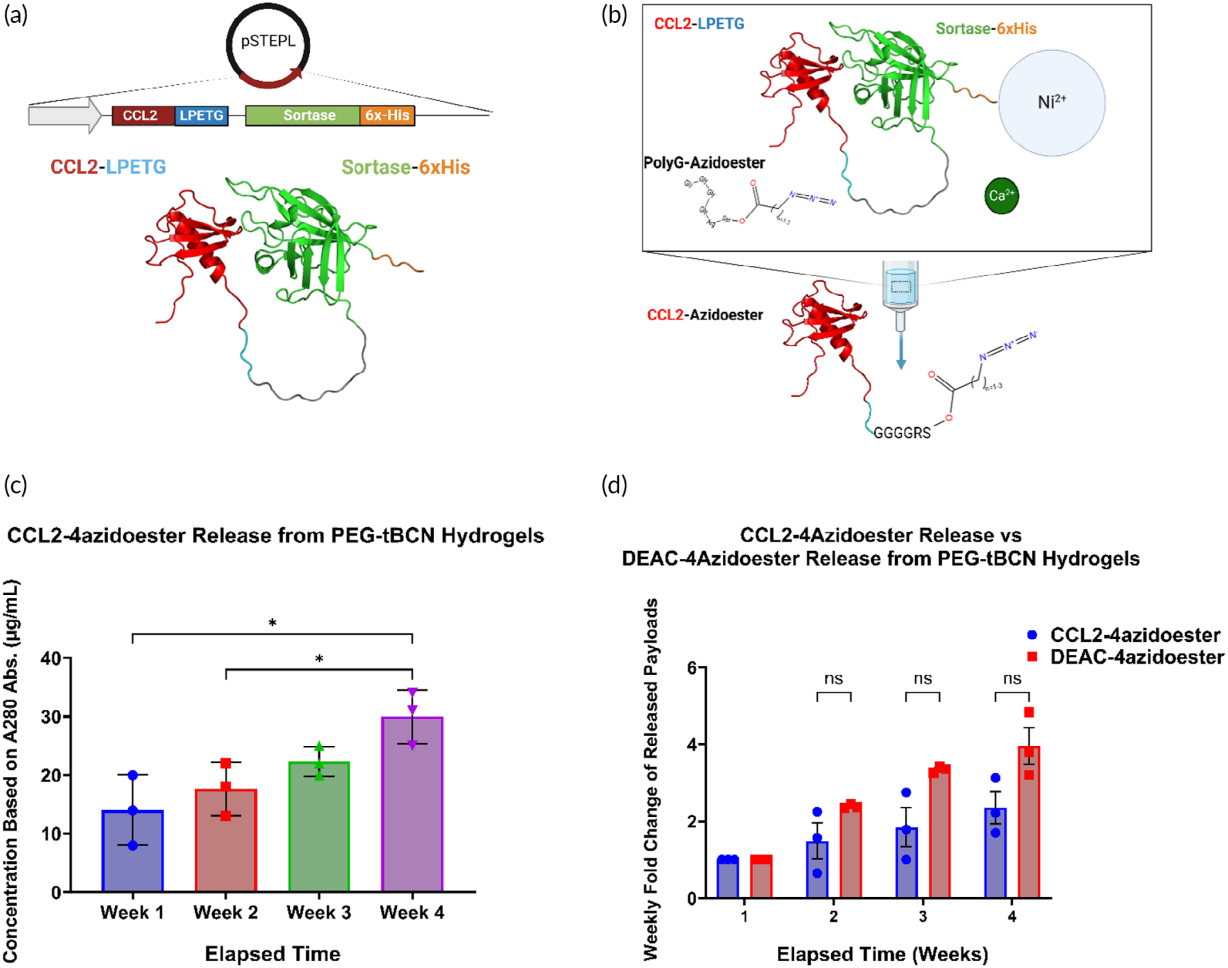
Hydrogel‐mediated chemokine gradients sustained via azidoester hydrolysis (A) This illustration depicts the structure of a CCL2‐STEPL fusion protein expressed via the STEPL system in E. coli.^92^, ^97^, ^98^, ^99^, ^100^, ^101^, ^102^ In this variant of sortagging, the protein of interest and sortase are co‐expressed on the same plasmid. (B) This illustration represents the STEPL process as follows: Purified fusion proteins are initially bound to a Ni‐NTA column. Upon adding PolyG‐azidoester and calcium, sortase catalyzes the simultaneous removal of CCL2 and the attachment of PolyG‐4Azidoester, resulting in CCL2‐4azidoester. This product can be collected in the flowthrough, while the remaining fusion protein continues to be bound to the Ni‐NTA column until elution.^103^ (C) This graph displays month‐long release profiles of CCL2‐4azidoester from a hydrogel in PBS at 37°C, quantified as μg/mL by *A*_280_ readings. Data are presented as mean ± SD for *n* = 3 replicates. Statistical significance was determined by one‐way ANOVA followed by Tukey's multiple comparison test. (D) In this graph, fold change of CCL2‐4azidoester release over 4 weeks is compared to that of DEAC‐4azidoester. Despite CCL2's much larger size, we observed no significant difference in release rates between the two species. Data are presented as mean ± SE for *n* = 3 replicates. Statistical significance was determined by multiple unpaired *t*‐tests followed by Holm–Šídák post‐hoc correction. [(ns) not significant, (*) *p* < 0.05.]

Concentration gradients are crucial for the function of some secreted immune cell‐enhancing proteins like chemokines, which drive directional cell movement along its gradient.^92^, ^93^, ^94^, ^95^, ^96^ Consistent azidoester hydrolysis over a desired timeframe offers a means to establish and maintain a chemokine gradient from a hydrogel source within tissue. We chose human C‐C motif chemokine ligand 2 (CCL2), a chemoattractant for monocytes and other immune cells, as a model protein for expression and PolyG‐azidoester transpeptidation in the STEPL bacterial expression system utilizing disulfide bond‐capable E. coli (Shuffle® T7 Express, New England Biolabs).^55^, ^92^, ^97^, ^98^, ^99^, ^100^, ^101^, ^102^ The overall principle of STEPL is the same as traditional sortase tagging in the sense of site‐specific transpeptidation of proteins with GGG‐containing molecules. However, in contrast to traditional sortase tagging employed in our previous experiment, where sortase and a protein of interest with a C‐terminal LPETG are expressed separately, the STEPL expression system creates a fusion between a protein of interest and sortase on the same plasmid (Figures 6a and S6). The reaction takes place on an affinity column where GGG‐modified proteins are liberated from the larger STEPL fusion protein in the flow‐through (Figure 6b). CCL2 within the STEPL plasmid was encoded with a single C‐terminal LPETG‐6xHis tag, “CCL2‐LPETG”. Consequently, a 1:1 protein‐to‐azidoester ratio from a successful sortase tag was expected to yield a release profile like DEAC‐OH (Figure 5d). In this regard, CCL2‐STEPL fusion proteins were reacted with the PolyG‐4azidoester and we collected our product “CCL2‐4azidoester” from the column flow‐through (Figures 6b and S7). Hydrogel solutions containing CCL2‐4azidoesters were permitted to react overnight before undergoing polymerization, casting, and immersion into PBS release media at 37°C. Weekly *A*_280_ measurements of the released protein demonstrated a consistent, quantifiable increase in detected protein in the release medium over a 4‐week period (Figure 6c). In our experiments, the fold change in CCL2‐4azidoester release each week did not significantly differ from the trend observed with DEAC‐4azidoester in Figure 5 over 4 weeks, despite their significantly different sizes (Figure 6d). This data suggests that hydrolysis of the azidoester is the limiting factor in the release of proteins of this size once tethered and released from the PEG‐tBCN hydrogel, rather than diffusion.

### 2.7 Hydrolytic release of CXCL10 gradients from intratumorally injected hydrogels induces T‐cell recruitment and attenuates growth in a syngeneic melanoma model

**FIGURE 7:**
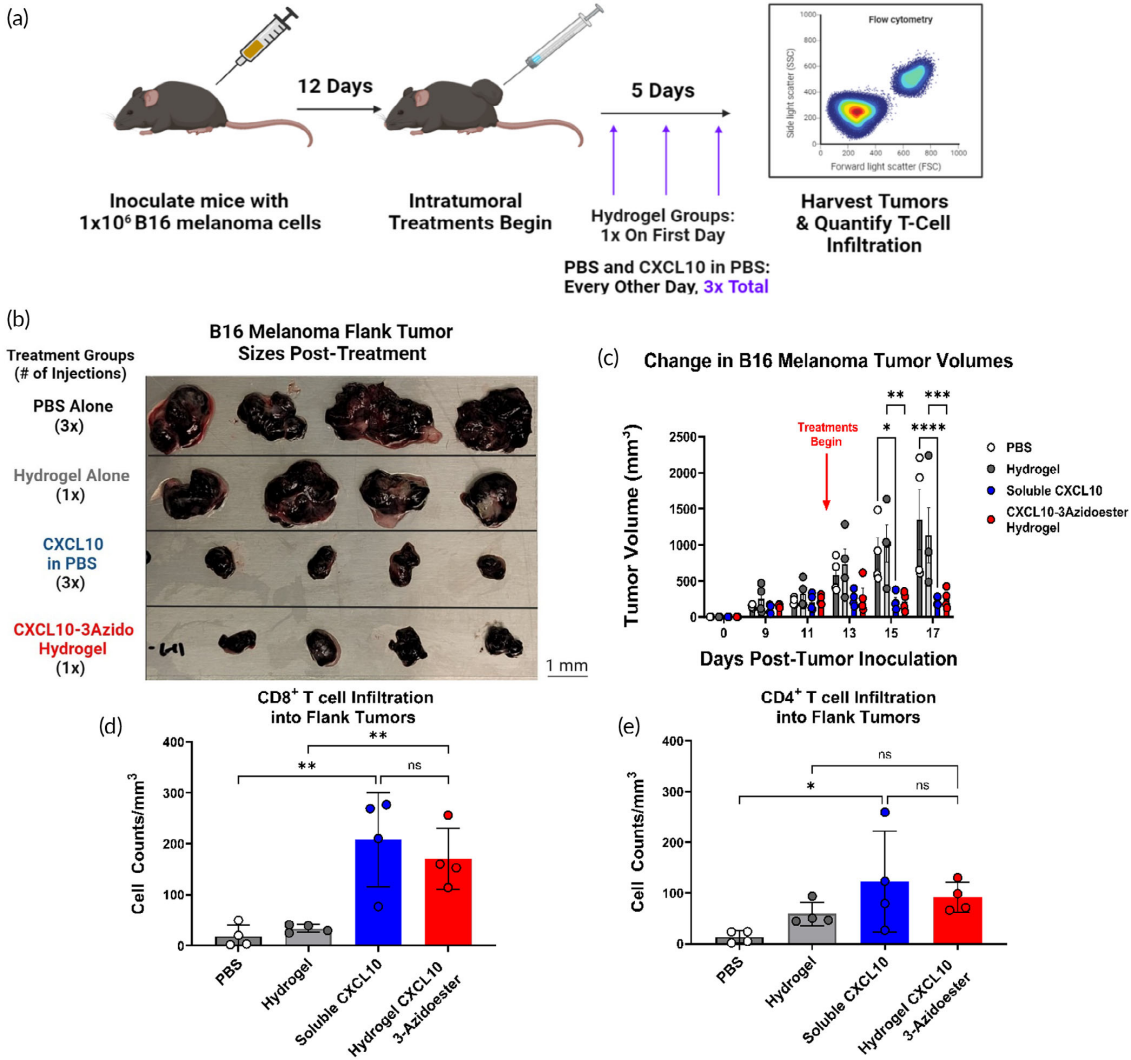
A single administration of a hydrogel housing mCXCL10‐3azidoester recruits T cells into a “Cold” Tumor. (A) This timeline illustrates the study design, including the timing of CD19^+^ B16 melanoma inoculations and the beginning of treatment administration: PBS Control, Hydrogel Control, Soluble mCXCL10, and mCXCL10‐3Azidoester Hydrogel.^106^, ^107^, ^108^, ^109^ (B) This image shows the sizes of the flank tumors collected at the end of the study and demonstrates a stark contrast between treatment groups. (C) Quantification of B16 melanoma tumor volumes over the course of the study reveals significant differences in mCXCL10 treatment groups vs their controls. Data are presented as mean ± SEM. Statistical significance was determined by two‐way ANOVA followed by Tukey's post‐hoc correction for multiple comparisons. (D) Quantification of CD8^+^ infiltrate into each tumor reveals a significant difference in CD8^+^ cells in tumors treated with both mCXCL10 treatments vs their control. Data are presented as mean ± SD for *n* = 4 replicates. Statistical significance was determined by one‐way ANOVA followed by Holm–Šídák post‐hoc correction. (E) Quantification of CD4^+^ infiltrate into each tumor reveals a significant difference in CD4^+^ cells in tumors treated with soluble mCXCL10 compared to its PBS control. Data are presented as mean ± SD for *n* = 4 replicates. Statistical significance was determined by one‐way ANOVA followed by Holm–Šídák post‐hoc correction. [(ns) not significant, (*) *p* < 0.05, (**) *p* < 0.01, (***) *p* < 0.001, (****), and *p* < 0.0001].

In this final in vivo study, we further demonstrate the translational potential of our hydrolysable release hydrogel system in a syngeneic “cold” mouse melanoma model to counteract T‐cell exclusion, which currently thwarts immunotherapy treatments in human patients.^104^, ^105^ We sought to utilize murine C‐X‐C motif chemokine ligand 10 (mCXCL10), a potent interferon γ‐induced T‐cell chemoattractant, as the bioactive protein of interest to potentially rectify this clinical problem.^106^, ^107^, ^108^, ^109^ Uncontrolled, systemic administration of this chemokine can potentially result in immune cell over‐activation and deadly cytokine release syndrome, so it would be another ideal immunotherapy candidate for local delivery into tissue, with our injected hydrogel deployed as the gradient source.^17^ We recombinantly expressed this murine CXCL10 (mCXCL10) in disulfide bond‐capable *E. coli* (Shuffle® T7 Express, New England Biolabs) with a C‐terminal LPETG‐6xHis tag, “mCXCL10‐LPETG” and employed traditional sortase tagging to modify it with PolyG‐3azidoester for PEG‐tBCN tethering and hydrolytic release (Figures S8 and S9). The PolyG‐3azidoester was chosen for mCXCL10‐LPETG linkage, “mCXCL10‐3azidoester”, because its expected hydrolysis rate of ~5%–6% per day, based on DEAC‐3azidoester data, was suitable for lasting the entirety of 5 days of treatment (Figures 5d and 7a). Chilled, 6.5% (w/v) hydrogel solutions were allowed to conjugate to 4.5 μg of mCXCL10‐3azidoester and were injected only once on day 1 of the treatment schedule. To match that estimated rate, we repeatedly administered 500 ng doses of mCXCL10 solutions in PBS every other day over the 5 days of dosing Upon completion of the study, we observed significant tumor growth attenuation and a significant increase in CD8^+^ T‐cell trafficking in B16 melanoma flank tumors after a single dose of the hydrogels tethered to mCXCL10‐3azidoester, equivalent to the results observed in the soluble mCXCL10 condition that was administered multiple times (Figures 7b–d and S10). Although both mCXCL10 treatment groups had a similar effect on CD8^+^ trafficking and attenuated tumor size, only the soluble mCXCL10 treatment resulted in a significant increase in CD4^+^ cells (Figures 7e and S10). Importantly, repeat administrations may not be practical in sensitive organs such as the brain, further supporting the use of a one‐time hydrogel application. PBS and hydrogel alone control groups showed no effect on T‐cell recruitment or tumor size, suggesting that the preserved biological activity of the chemokines produced the observed results. Thus, we demonstrate another practical scenario in which locally delivering a bioactive, protein‐based therapeutic from a single dose of PEG‐tBCN hydrogel may be favorable.

## 3 DISCUSSION

Our study aimed to develop versatile, highly customizable bioorthogonal PEG hydrogels for safe and effective loco‐regional delivery of protein therapeutics. This system is particularly useful for proteins with known systemic toxicities that would benefit from local, controlled release, like many immunotherapies. These hydrogels simplify many clinical requirements into one platform, offering user‐defined release capabilities, injectability into tissue, and adaptability to a variety of payloads with differing clinical utilities, including the delivery of checkpoint blockers or the generation of immune cell chemoattractant gradients. The first clinical requirement we prioritized was a high degree of biocompatibility with tissue, and this is reflected in the construction of our material's backbone and polymerization chemistry. PEG, a nontoxic and lowly immunogenic polymer, is currently employed in multiple FDA‐approved applications, including therapeutics such as Neulasta and Movantik.^110^, ^111^ Our PEG‐based hydrogel is polymerized with SPAAC chemical handles, which is an catalyst‐free variant of azide‐alkyne click chemistry.^51^ SPAAC is a nontoxic click reaction that has demonstrated safety in the presence of living cells and enhances the promise of our hydrogel system for clinical applications.^35^, ^36^, ^37^, ^112^ Furthermore, SPAAC reactions have shown promise in the construction of antibody drug conjugates utilized in clinical trials, including STRO‐001 and ADCT‐601.^113^, ^114^ As demonstrated in our study, one major benefit of our tissue‐injectable hydrogel is the minimally involved ability to deposit hydrogel solutions into subsurface locations (Figures 1, 2 and S1). This feature may be essential in locations like the brain, where extensive surgeries to create a resection cavity could cause unacceptable harm to the patient. In contrast to our system, many preclinical studies showcasing novel hydrogel formulations and delivery modalities tend to utilize artificially created resection cavities or superficially located flank tumors.^38^, ^39^, ^40^, ^41^, ^42^, ^43^, ^44^, ^45^, ^46^ Clinicians already have the ability to insert solid polymers into superficial tumors or resection cavities.^4^, ^5^, ^49^ Therefore, we believe our system is uniquely capable of delivering protein therapeutics in a location‐agnostic fashion.

This system was developed with broad versatility in mind, enabling the incorporation of various methods for modifying and transiently linking bioactive protein payloads to the hydrogel backbone. This flexibility is essential if the gel is to be used as a delivery depot for combinatorial therapeutics with differing physiochemical properties. SPAAC and sortase‐mediated bioconjugation strategies that we employed can be substituted or complemented with alternative click chemistry ligation methods, diverse protein modification techniques, and various mechanisms for protein‐linker cleavage. Other click reactions, such as oxime ligation, tetrazine ligation, nitrone dipole cycloaddition, and tetrazole photochemistry, can be used alone or possibly in combination with SPAAC reactive handles based on preference.^115^, ^116^, ^117^, ^118^, ^119^, ^120^, ^121^ Enzymatic bioconjugation strategies, such as SpyTag/SpyCatcher and HaloTag, are among some of the site‐specific protein modification techniques that can be used to complement, or substitute, “sortagging” to conjugate therapeutic proteins to adapter peptides like H‐GGGGRS‐NH_2_ (Figure 3).^33^, ^122^, ^123^, ^124^, ^125^ In fact, through genetic code expansion, noncanonical amino acids with chemical handles may be directly translated into a protein of interest, forgoing the need for polypeptide adapters entirely, if desired.^89^, ^122^, ^126^ Overall, this system's flexibility in accommodating a more complex set of polymerization and protein linkage modalities could allow for multiple site‐specific protein modifications that need to be orthogonal to each other to maintain protein bioactivity. For instance, as demonstrated with αCD47‐LPETG, two azides can be conjugated onto one protein, facilitating the possibility of heterogenous modifications (Figure 4). This versatility can be particularly useful for “knob in holes” bispecific immune cell engagers, enabling a 1:1 protein‐to‐linker ratio on one sortase motif and completely different payload conjugations to the other motif(s).^127^

We believe that affording extended‐release capabilities to our hydrogel may be essential in some clinical contexts, particularly for proteins that have short tissue half‐lives or those whose functions rely on concentration gradients. This approach avoids the need for multiple surgeries and helps mitigate the toxicities associated with a burst release of drugs into tissue.^4^, ^5^, ^48^, ^128^ One can achieve some degree of release control simply by adjusting the gel's crosslink density. The average pore size of the gel network is dependent on the length of the PEG chains and is directly related to diffusion rates out of the gel.^25^, ^48^, ^112^, ^129^ However, it is important to recognize that adjusting the diffusion rate of protein payloads, may only extend release rates on the scale of hours.^112^ In our hands, adjusting our gel's mesh size still resulted in rapid diffusion of large antibodies within 24 h (Figure S3). Unlike an IgG, which can persist in the body for many days without user manipulation, this may not be sufficient for therapeutics with short tissue half‐lives.^130^, ^131^, ^132^, ^133^ In contrast to our system, the FDA‐approved Oncogel polymer, for example, can sustain the release of paclitaxel over several months, similar to the release rate achieved with DEAC‐OH on the 4‐azidoester linker (Figure 5).^48^ However, this prolonged diffusion rate primarily results from interactions between the hydrophobic drug and the co‐block polymer, a situation not applicable to hydrophilic protein therapeutics within a hydrogel. Another strategy employed clinically to extend the release of therapeutics is the slow physical degradation of the polymer to release payloads, as seen in the FDA‐approved Gliadel polymers. Nonetheless, this approach can affect drug distribution profiles since the polymer does not maintain the same contact with the tissue over time.^4^, ^5^, ^49^, ^134^, ^135^ We believe our hydrolytic linker strategy is the superior option compared to these FDA‐approved methods for maintaining long‐term, consistent protein release from an implanted depot. Our system uniquely provides the ability to bestow a range of predictable release rates to protein or small molecule therapeutics, if desired. We envision a scenario where clinicians can generate a hydrogel solution patterned with therapeutic combinations via appropriately sized azidoesters that match their differing effective concentrations, function, and tissue half‐lives.

If concerns arise regarding the foreign body response to our nondegradable hydrogel (as currently designed), we propose a straightforward method to confer depolymerization capabilities *after* payload delivery. This approach directly addresses the limitations of the degradable Gliadel polymers. Initially, we conjugated azido acids to linear PEG‐NH_2_ through NHS‐ester chemistry to create amidated PEG‐Diazide crosslinkers (Figure 1). However, by substituting linear PEG‐NH_2_ with PEG‐OH, we can synthesize “PEG‐Diazidoester” crosslinkers using carbodiimide chemistry (Figure S11). These esterified crosslinkers have the potential to hydrolyze at a predictable rate in aqueous solutions based on the original azidoacid length, resulting in gradual gel network depolymerization over time. Notably, there are longer linear ω‐terminal azido acids available than those employed in our study (e.g., azido acids with ≥5 carbon atoms), which could enable complete gel breakdown over significantly longer timeframes compared to what we observed for payload release. Lastly, although our hydrogel system provides a simple “fire‐and‐forget”, method of protein release, this mechanism can be more tightly controlled by substituting hydrolytic azidoesters with light‐responsive, drug‐sensitive, or Boolean‐logic linkers.^29^, ^30^, ^31^, ^32^, ^122^, ^136^ In summary, this highly versatile hydrogel‐based, local delivery system has the potential to be a broadly adaptable solution for a wide range of clinical requirements and scenarios.

## 4 MATERIALS AND METHODS

### 4.1 Synthesis and validation of hydrogel components

#### 4.1.1 PEG‐tetraBCN hydrogels

The individual hydrogel components used in these studies include: (1R,8S,9s)‐bicyclo[6.1.0]non‐4‐yn‐9‐ylmethyl (2,5‐dioxopyrrolidin‐1‐yl)carbonate (BCN‐OSu), poly(ethylene glycol) tetrabicyclononyne (PEG‐tetraBCN, *M*_n_ ~ 20,000 Da), 2,5‐dioxopyrrolidin1‐yl 4‐azidobutanoate (N_3_‐OSu), and poly(ethylene glycol) diazide (PEG‐diazide, *M*_n_ ~ 3400 Da). These components were synthesized and polymerized into PEG‐tetraBCN hydrogels as previously reported in greater detail, including characteristics such as G′ and G″.^29^, ^32^, ^36^ In brief: 4‐arm PEG‐OH was functionalized with BCN‐OSu (BCN‐NHS ester) at a 1:4 molar ratio to create the PEG‐tetraBCN gel backbone. Linear PEG‐OH was functionalized with N_3_‐OSu (N_3_‐NHS ester) at a 1:2 ratio to create the hydrogel crosslinker, PEG‐diazide. Hydrogels are polymerized by mixing PEG‐tetraBCN backbones with PEG‐diazide at 1:4 molar ratios at 37°C. Fully polymerized gels were formulated at concentrations of 8% (w/v) for in vitro studies and 6.5% (w/v) for in vivo injections, as this concentration was easier to aspirate with a Hamilton syringe.

#### 4.1.2 DEAC 2,3,4‐azidoester synthesis and DEAC‐Azidoester hydrolysis from PEG‐tetraBCN hydrogels

7‐(Diethylamino)‐4‐(hydroxymethyl)coumarin (DEAC‐OH) was previously synthesized in‐house (Method S3) and conjugated to 2‐azidoacetic acid (Click Chemistry tools, 1081), 3‐azidopropionic acid (Synthonix, A1939), and 4‐azidobutyric acid (Synthonix, A1941) via Steglich esterification. Briefly, 0.13 mmol of DEAC‐OH, 0.30 mmol of DMAP, and 0.13 mmol of the corresponding azido acid were mixed in minimal DCM and stirred for 10 minutes at room temperature. Next, 0.15 mmol of EDAC was added to the mixture, and the reaction was stirred overnight. The completed reaction was passed through a vacuum filter to remove urea byproducts and dried under vacuum. To prepare the hydrogel, 100 μM DEAC‐azidoester conjugates were suspended in DMSO and “clicked” onto the backbone of PEG‐tetraBCN hydrogel solutions overnight before polymerization. The resulting gels were cast as 10 μL cylinders and plated in triplicate into a 12‐well plate containing 500 μL PBS/well. The plate was incubated at 37°C and 5% CO_2_ for the duration of the experiment. At each timepoint, supernatants were collected from each well, and the fluorescence of the released DEAC‐OH was measured using a Molecular Devices SpectraMax plate reader (Laser line 405; *λ*_ex_ 387, *λ*_em_ 470 nm). The concentration of DEAC‐OH was determined using linear regression of a DEAC‐OH standard in PBS.

#### 4.1.3 GGGGRS‐3,4‐azidoester (PolyG‐azidoester)

The Fmoc‐GGGGRS‐NH_2_ polypeptide, which had C‐terminal amidation, was either synthesized in‐house (Method S1–S2), or purchased from Biomatik Corporation (Ontario, CA). Fmoc‐GGGGRS 3,4‐azidoesters were produced using Steglich esterification, a type of carbodiimide chemistry.^71^ In particular, 0.45 mmol of Fmoc‐GGGGRS, 2.0 mmol of DMAP (Sigma‐Aldrich, 851,055), and 0.74 mmol of 3 or 4 azidoacids were stirred for 10 minutes at 40°C in minimal dimethylformamide (Sigma‐Aldrich, 319937). The solution was then added with 0.74 mmol of DCC (Sigma‐Aldrich, D80002) and stirred overnight at 40°C. Following this, the polypeptide‐azidoester was Fmoc‐deprotected by adding piperidine (ChemImpex, 02351) to a final concentration of 20% and stirred for 5 minutes. Crude polypeptide‐azidoester was then precipitated in cold di‐ethyl ether, HPLC purified using 95:5 H_2_O/acetonitrile, and lyophilized. The completed products, with an appearance of a clear‐yellow oil, was confirmed via MALDI‐TOF mass spectrometry (Figure S5). The purified “PolyG‐azidoesters” were stored at −20°C under a nitrogen atmosphere for future use.

### 4.2 Expression and purification of recombinant proteins

#### 4.2.1 αCD47‐LPETG

The Heavy and Light chain amino acid sequences of the 2.3D11 clone of the CD47mAb was obtained from US Patent US 9,650,441 B2. These sequences, along with CH2‐CH3 of the human IgG1 constant region were inserted into the pCVL‐SFFV‐muScn‐IRES‐GFP mammalian expression plasmid (Genscript, Nanjing, Jiangsu, China). Protein was expressed utilizing the Daedalus system with a C‐terminal LPETG—6xHis tag. Proteins were purified and stored in PBS for later use (Figure S4).^84^

#### 4.2.2 CCL2‐LPETG

The mature amino acid sequence for human CCL2 (aa 24–99) was obtained from NCBI, GeneID 6347. gBlocks (IDT) were created for this sequence with a C‐terminal “LPETG” Sortase recognition site and complementary 5′ and 3′ overhangs to the NdeI/XhoI double digested pSTEPL Sortase fusion expression plasmid.^55^ The resulting gBlocks were ligated into pSTEPL plasmids using Gibson assembly (NEB) and transformed into chemically competent SHuffle® T7 Express E.coli (NEB). After bacterial liquid cultures were grown to 0.6 OD_600_, protein expression was induced overnight at 16°C with 0.2 mM IPTG (Thermofisher, 15529019). The overnight cultures were lysed and sonicated in non‐denaturing conditions: 20mM Tris, 125mM NaCl, 10mM imidazole, 0.1% Triton X‐100 + COmplete tablet (Millipore Sigma, 11873580001), and the CCL2‐LPETG STEPL fusion proteins were purified via Ni‐NTA pulldown for PolyG‐azidoester modification (Figures S6 and S7).

#### 4.2.3 mCXCL10‐LPETG

The mature amino acid sequence of murine CXCL10 (aa 22–98) was obtained from NCBI, GeneID 15945. gBlocks were created for this sequence with a C‐terminal “LPETG” Sortase recognition site and complementary 5′ and 3′ overhangs to the BamHI/HindIII double digested pCARSF63 Thioredoxin‐SUMO fusion expression plasmid (Addgene #64695).^137^ The resulting gBlocks were ligated into pCARSF63 expression plasmids using Gibson assembly (NEB) and transformed into chemically competent SHuffle® T7 Express E.coli (NEB). After bacterial liquid cultures were grown to 0.6 OD_600_, protein expression was induced overnight at 16°C with 0.2 mM IPTG. The overnight cultures were lysed and sonicated in nondenaturing conditions: B‐PER Complete (Thermofisher, 89821), 10 mM imidazole, 0.1% Triton X‐100 + COmplete tablet (Millipore Sigma, 11873580001) on ice. mCXCL10‐LPETG SUMO fusion proteins were purified by Ni‐NTA pulldown and then treated overnight with Endotoxin Removal columns (Thermofisher, 88274). Cleavage of mCXCL10‐LPETG from the greater SUMO fusion proteins was carried out via ULP1 digestion (Thermofisher, 12588018) and stored for subsequent PolyG‐azidoester modification (Figures 7 and S8).

#### 4.2.4 Sortase 5M

Bacterial stabs containing Sortase 5M were obtained from Addgene (#51140), deposited by the Ploegh lab.^138^ Individual colonies were picked from plated stabs for 10 mL plasmid preps (Invitrogen, K210010), and purified plasmids were transformed into chemically competent T7 Express E. coli (NEB). Cultures were induced with 0.2 mM IPTG overnight at 16°C, lysed and sonicated on ice in nondenaturing conditions: 20 mM Tris, 125 mM NaCl, 10 mM imidazole, 0.1% Triton X‐100 COmplete tablet (Millipore Sigma, 11873580001). Sortase 5 M proteins were purified by Ni‐NTA pulldown and stored for later use in αCD47‐LPETG, and mCXCL10‐LPETG sortase tagging experiments.

### 4.3 Sortase tagging and modifying recombinant proteins

#### 4.3.1 αCD47‐4azidoester

20 μM αCD47‐LPETG, 10 μM sortase 5 M, and 10 mM CaCl^2+^ were mixed with 500 μM of PolyG‐4azidoester or 500 μM triglycine (GGG) control in sortase reaction buffer (20 mM Tris, 50 mM NaCl, pH 7.5) for 4 h at 37°C. Ni‐NTA resin was added to remove any unreacted antibody and sortase 5 M. The supernatant, containing pure αCD47‐4azidoester, was collected, spin‐concentrated with 50 kDa MWCO columns, and buffer‐exchanged into PBS.^54^, ^84^ Bound IgG was eluted and collected for western blot. Separately, 5 μM of purified αCD47‐LPETG and αCD47‐4azidoester were mixed with 140 μM DBCO‐IR Dye 680 (Licor, 929‐50005) at 37°C shaking at 500 rpm overnight. Excess dye was washed away with repeated buffer exchanges into to PBS and concentration in 50 kDa MWCO spin columns (Figure 4).^54^, ^80^

#### 4.3.2 CCL2‐4azidoester

Ni‐NTA resin‐bound STEPL fusion proteins containing CCL2‐LPETG were incubated with 1 mM PolyG‐4‐azidoester in sortase reaction buffer for 4 h at 37°C while shaking. Successful sortase tagging releases CCL2‐LPETG from the greater fusion protein, leaving the remaining fusion protein bound to Ni‐NTA resin. Pure CCL2‐4azidoester was collected in the column flow through, concentrated using 3 kDa molecular weight cut‐off (MWCO) columns, and buffer‐exchanged into PBS (Figures 6 and S7).^55^

#### 4.3.3 mCXCL10‐3azidoester

50 μM of ULP1‐digested chemokine was reacted with 10 μM of sortase 5 M and 500 μM of PolyG‐3‐azidoester in sortase reaction buffer for 4 h at 37°C. Ni‐NTA resin was added to remove any unreacted chemokine and sortase 5 M. The supernatant, containing pure mCXCL10‐3‐azidoester, was collected, spin‐concentrated with 3 kDa MWCO columns, and buffer‐exchanged into in vivo‐grade PBS (Figures S8 and S9).^54^

### 4.4 Animal and cell line acquisition

#### 4.4.1 Athymic Nu‐/Nu‐ (Harlan) and C57BL/6 mice

Mice used in this study were purchased from the Jackson Laboratory (Bar Harbor, ME, USA). Female Athymic *Nu‐/N*u‐ (Harlan) and female C57BL/6 mice were used in the presented data. We have not observed sex to play a role in biological outcomes. All animals were used in accordance with FHCC and SCRI Institutional Animal Care and Use Committee guidelines, protocols 1457 (FHCC) and 00106 (SCRI).

#### 4.4.2 B16.F10 melanoma cells

B16.F10 cell lines were obtained from ATCC. These lines were expanded from single clones transduced with lentivirus to express EGF, huCD19, and firefly Luciferase. The sex of the cell lines has not been documented.

#### 4.4.3 Patient‐derived xenograft (PDX) pHGG “PBT05”

PBT05 cells were obtained from a female patient's biopsy (Seattle Children's Hospital/Children's Oncology Group) and cultured in Neuralcult NS‐A Basal Medium (STEMCell Technologies) with Proliferation supplement (STEMCell, 05753), PenStrep (Thermofisher), Glutamax (Thermofisher) EGF (PeproTech, AF‐100‐15) and FGF (PeproTech, 100‐18B). Cells were grown adherent on tissue‐culture treated plates after at least 2 h of Laminin coating (Sigma‐Aldrich) in an incubator at 37°C in 5% CO_2_. All PDX lines were lentivirally transduced with GFP or mCherry/Luciferase to assist in Incucyte cell counting and tumor size visualization via IVIS imaging.

#### 4.4.4 Murine macrophages

Murine monocytes were harvested and cultured from femurs of C57BL/6 mice using RPMI (Thermofisher, 11875093) containing 10% heat deactivated FBS and 100 ng/mL mCSF1 for 7 days. Mature macrophages from these cultures were later harvested for co‐culture experiments.

### 4.5 In vitro and in vivo hydrogel studies

#### 4.5.1 AF594 hydrogel diffusion assay

200 μL master mixes of PEG‐tBCN gels ranging from 5.2% to 8% (w/v) were formulated, admixed with 1 mg/mL of Goat anti‐mouse AF594 conjugates (Thermofisher A‐11032) or with an equivalent volume of PBS. Gels were cast in triplicate in 1.5 mL Eppendorf tubes and left overnight at RT under foil. A standard curve was generated of the antibody in serial dilutions in PBS using the Nanodrop One “Protein and Labels” quantification feature (Thermofisher) and was used to calculate fraction released from each gel group (Figure S7).

#### 4.5.2 Cortical hydrogel injection

Pre‐silanized Hamilton Neuros Syringes (Hamilton, 65460‐06) were used to aspirate 3 μL of chilled 6.5% (w/v) PEG‐tetraBCN hydrogel solutions (PEG‐tBCN backbone with PEG‐Diazide crosslinker). The solution was promptly injected ~1–3 mm deep into the parenchyma of the cortex of C57BL/6 mice anesthetized with isoflurane. Buprenorphine SR was used as analgesia. After injection, mice were allowed to recover and returned to group housing with no limitations on mobility or access to food and water. Mice were euthanized 7 days after implant and, brains were fixed in 4% neutral‐buffered formalin and prepared for IHC. This experiment was repeated dozens of times, with representative images from *n* = 3 mice included within the text (Figures 1 and S1).

#### 4.5.3 Patient‐derived xenograft (PDX) pHGG, PBT05, cortical inoculation and treated hydrogel administration

Luciferase^+^ PDX tumors were established in the cortex of 15 female Athymic *Nu‐/Nu*‐ (Harlan) mice. Inclusion criteria: intracranial tumors were allowed to grow to z flux value of 1e^7^ before study enrollment, and mice were sorted into treatment groups using a random number generator. Pre‐silanized Hamilton Neuros Syringes (Hamilton, 65460‐06) were used to aspirate 3 μL of chilled 6.5% (w/v) PEG‐tetraBCN hydrogel solutions (PEG‐tBCN backbone with PEG‐Diazide crosslinker) admixed with either 2.6 μg of αCD47‐LPETG or admixed 2.6 μg of BioXcell B6H12 anti‐CD47mAb. The solution was promptly injected ~2 mm deep into the parenchyma of the cortex of pre‐PDX inoculated mice anesthetized with isoflurane. Buprenorphine SR was used as analgesia. After injection, mice were allowed to recover and returned to group housing with no limitations on mobility or access to food and water. Mice were monitored for the duration of the experiment for luminescence tracking via IVIS. Upon study completion, mice were euthanized, and their brains were fixed in 4% neutral‐buffered formalin and prepared for IHC (Figure 2).

#### 4.5.4 B16 Melanoma Flank tumor inoculation and treatment administration

1 × 10^6^ tumor cells were injected subcutaneously on the right flank of 20 Female C57BL/6 mice. Inclusion criteria: mice with established tumors between 150 and 400 mm^3^ were included in the study. Mice were distributed into groups of *n* = 4–5 to normalize mean tumor volume and standard deviation prior to treatment which was randomly assigned by the Rand() function in Microsoft Excel. Half of the mice were injected intratumorally every other day with murine CXCL10 in PBS (PeproTech, Cranbury, NJ) or a PBS vehicle control. The other half of the mice were injected once with mCXCL10‐3azidoester‐containing hydrogel or an empty hydrogel control. Total injection volumes of 20 μL were used for each treatment group (Figure 7).

### 4.6 Tissue, western blot, and cell culture analysis

#### 4.6.1 Locating injected gels in brains via histology

Mouse brains were harvested, formalin fixed, and paraffin embedded. Brain blocks were then sliced and stained with H&E. Brain sections were imaged using a TISSUEFAX slide scanner (Gnosis) in the imaging core at FHCC (Figures 1, 2 and S1).

#### 4.6.2 Flow cytometry antibodies

Antibodies and live cell dyes used in the PBT‐05 in vitro analysis and B16 melanoma flank tumor analysis were procured from eBiosciences (San Diego, CA), Jackson ImmunoResearch Labs (West Grove, PA, USA), or Biolegend (San Diego, CA, USA). The antibodies included APC Anti‐Human CD47 B6H12 clone (eBiosciences, 17‐0479‐42), APC Murine IgG1 Isotype Control (Biolegend, MOPC‐21), APC Human IgG1 Isotype Control (Biolegend, 403505), APC Goat Anti‐Human IgG 2° (Jackson), APC Goat Anti‐Mouse IgG 2° (Jackson), Zombie AquaTM live dead (Biolegend), CD8a (53‐6.7, Biolegend), CD4 (RM4‐5, Biolegend), and CD3 (17A2, Biolegend). 5 × 10^6^ cells were stained for surface or intracellular proteins by incubating cells with antibodies diluted in PBS + 2% BSA for 45 minutes on ice. Cells were then washed 3× in flow cytometry stain buffer and fixed with 2% PFA for 20 minutes prior to acquisition on a LSRII Fortessa (BD Biosciences, San Jose, CA, USA). Samples were analyzed with FlowJo V10 software (Figures 7 and S10).

#### 4.6.3 Quantifying B16 tumor volume and T‐cell infiltration

Tumor measurements via caliper began 7 days post‐tumor cell injection and were carried out every 2–3 days afterward. Tumor volumes were calculated using the formula (length × width^2^)/2. At the conclusion of the study, tumors were homogenized into single cell suspensions, stained with a live/dead viability marker and fluorescently conjugated antibodies for CD8, CD4, CD3, and CD44. Cells were analyzed via flow cytometry and total cell counts were calculated by multiplying the target population (i.e., CD8^+^) frequencies of total viable cells by total hemacytometer cell counts. Cell counts were normalized to tumor volume by dividing target populations by tumor volume (Figures 7 and S10).

#### 4.6.4 In vitro phagocytosis assays

LEAF hCD47mAb was purchased from BioXcell (BE0019), R848 was purchased from Sigma Aldrich (SML0196) and murine IFNg was purchased from PeproTech (315‐05). Phagocytosis assays were performed using the Basic Analyzer software on Essen Bio/Sartorius Incucyte Zoom and Incucyte S3. A total of 12 or 24 well plates were seeded 1:1 with GFP^+^ PDX cells and bone marrow‐derived murine macrophages in fully supplemented Neuralcult plus aforementioned immunostimulatory molecules. Using custom counting definitions set to identify GFP^+^ nuclei, the Incucyte calculated tumor cell counts based on the number of GFP^+^ nuclei in the wells over time (Figure S2).

*Western Blot Analysis* Western blots were run using 4%–12% Bis‐tris gels (Fisher Sci) in MES running buffer at 180 V for 30 minutes. Gels were transferred to nitrocellulose using the Iblot or the Iblot 2 semi‐dry transfer devices (Fisher Sci). Antibodies and dyes used in this study were: Anti‐Human IgG Fc specific (Millipore Sigma I2136‐1ML), Anti‐6XHis tag (Abcam ab9108), Human CCL2/JE/MCP‐1 Antibody (RnD Systems, F‐279), IRDye 680‐DBCO (Licor, 929‐50005), Various 680 and 800 channel secondaries (Licor). Blots were scanned and analyzed on the Licor odyssey scanner and software (Figure 4).

#### 4.6.5 Bioluminescence imaging

Mice harboring Luciferase^+^ orthotopic xenografts (PBT05) were injected IP with D‐Luciferin (Xenolight) at concentrations of 3 mg/100 μL PBS per mouse. 3 min post injection of D‐Luciferin, mice were anesthetized using isoflurane for an additional 7 minutes. 10 minutes post‐Luciferin injection, anesthetized mice were placed in the IVIS (Perkin Elmer) chamber and bioluminescence imaging was obtained with 1 minute exposure time, F/stop 1 and 8, field D. Luminescent photos and total flux ROIs was analyzed using Living Image software (PerkinElmer) (Figure 2).

### 4.7 Statistical analyses

Statistical analyses were performed using GraphPad Prism version 9, licensed by SCRI (GraphPad Software, CA, La Jolla, USA). All error bars are specified as either the mean ± standard deviation (SD) or mean ± standard error of the mean (SEM). Hypothesis tests used in this study were: Multiple unpaired *t*‐tests with Holm–Šídák post‐hoc correction for multiple comparisons, One‐Way ANOVA with Tukey's post‐hoc correction for multiple comparisons, and two‐Way ANOVA with Holm–Šídák or Tukey's post‐hoc correction for multiple comparisons. *p*‐values <0.05 were considered statistically significant.

## 5 CONCLUSIONS

In conclusion, our study demonstrates the successful development of a highly customizable platform for local protein delivery that combines bioorthogonal PEG hydrogels and site‐specific protein conjugation via azidoester linkage. Our results show that by modifying the length (hydrophobicity) of the azidoester linker, the release rate of the protein payload can be tailored to suit a range of clinical needs. We also showcase two distinct protein conjugation strategies utilizing the sortase enzyme, Traditional and STEPL, which are amenable to a variety of therapeutically relevant proteins and expression systems, including in‐house expressed anti‐CD47 antibodies and murine CXCL10. Importantly, sortase modification and gel inclusion processes do not disrupt the biological function of the proteins.

The SPAAC‐based hydrogel system is injectable, polymerizes rapidly in situ, and can be located within tissue days after injection with no noticeable effect on the health of the animal. These features make it particularly well‐suited for small, sensitive spaces such as the brain, or for tumors that are not localized to superficial locations. Our study demonstrates this platform can deliver recombinant anti‐CD47 monoclonal antibodies into sub‐surface xenograft high‐grade glioma tumors and observe bioactivity comparable to a commercially available alternative. Additionally, murine CXCL10‐3azidoester delivered from one hydrogel injection was successful at recruiting significantly more CD8^+^ T‐cells and attenuating tumor growth in a cold melanoma tumor model compared to controls. Thus, we highlight two distinct preclinical scenarios where our hydrogel was deployed to deliver soluble, bioactive proteins with differing functions and achieve efficacy.

Overall, our work brings together the fields of chemical engineering, protein engineering, and biology to create a versatile and modifiable platform that can address a range of clinical needs. This platform has the potential to improve current methods of local protein delivery and we believe it will be of great interest to the scientific community working in this field.

## AUTHOR CONTRIBUTIONS

**Eric S. Nealy:** Conceptualization; data curation; formal analysis; investigation; methodology; validation; visualization; writing – original draft; writing – review and editing. **Steven J. Reed:** Data curation; formal analysis; investigation; methodology; writing – review and editing. **Steven M. Adelmund:** Formal analysis; investigation; methodology; writing – review and editing. **Barry A. Badeau:** Formal analysis; investigation; methodology; writing – review and editing. **Jared Shadish:** Formal analysis; investigation; methodology; writing – review and editing. **Emily J. Girard:** Data curation; formal analysis; investigation; methodology; writing – review and editing. **Kenneth Brasel:** Investigation; methodology; resources; supervision. **Fiona J. Pakiam:** Data curation; formal analysis; investigation; methodology; visualization. **Andrew J. Mhyre:** Conceptualization; supervision; writing – review and editing. **Jason P. Price:** Data curation; methodology; resources. **Surojit Sarkar:** Conceptualization; project administration; resources; supervision. **Vandana Kalia:** Conceptualization; project administration; resources; supervision. **Cole A. DeForest:** Conceptualization; funding acquisition; project administration; resources; supervision; writing – original draft; writing – review and editing. **James M. Olson:** Conceptualization; funding acquisition; project administration; resources; supervision; writing – original draft; writing – review and editing.

### PEER REVIEW

The peer review history for this article is available at https://www.webofscience.com/api/gateway/wos/peer-review/10.1002/btm2.10668.

## Supporting information


**Data S1.** Supporting Information.

## Data Availability

The data that support the findings of this study are available from the corresponding author upon reasonable request.
