# Supplementary material for: Versatile tissue‐injectable hydrogels capable of the extended hydrolytic release of bioactive protein therapeutics
Source: Bioeng Transl Med. 2024 Apr 15;9(5):e10668. doi: 10.1002/btm2.10668 (PMC11561820; doi:10.1002/btm2.10668)
Supplement: Supplementary file 1 — Data S1. Supporting Information. [file BTM2-9-e10668-s001.docx]

Supplementary Information for

**Versatile Tissue-Injectable Hydrogels Capable of Extended Hydrolytic Release of Bioactive Protein Therapeutics**

Eric S. Nealy, Steven J. Reed, Steven M. Adelmund, Barry A. Badeau, Jared A. Shadish, Emily J. Girard, Kenneth Brasel, Fiona J. Pakiam, Andrew J. Mhyre, Jason P. Price, Surojit Sarkar, Vandana Kalia, Cole A. DeForest^*^, James M. Olson^*^

*Corresponding authors. Email: [profcole@uw.edu](mailto:profcole@uw.edu) and [Jim.olson@seattlechildrens.org](mailto:Jim.olson@seattlechildrens.org)

Table of Contents

General Synthetic Information 3

Synthesis of Previously Reported Hydrogel Compounds Used in this Work 4

Method S1 Fmoc Solid-Phase Peptide Synthesis 5

Method S2 Synthesis of Fmoc-GGGGRS-NH_2_ 6

Method S3 Synthesis of DEAC-OH 7

Figure S1 Intracortical Hydrogel Injections in Mouse Brains *in vivo* 8

Figure S2 Anti-CD47 Antibody Blockade Results in Phagocytosis of Human pHGG Xenograft Cells by Murine Macrophages 9

Figure S3 IgG-AF594 Diffusion from 5.2-8% (w/v) PEG-tBCN Hydrogels 10

Figure S4 Expression of “αCD47mAb-LPETG” IgG1 with C-terminal Sortase Motifs 11

Figure S5 MALDI-TOF Confirmation of PolyG-3,4azidoester Synthesis 12

Figure S6 Amino Acid Sequence of Human CCL2 STEPL Fusion Protein 13

Figure S7 Conjugating PolyG-4azidoester to Human CCL2 via STEPL 14

Figure S8 Amino Acid Sequence, Model and Processing of mCXCL10 SUMO Fusion 15

Figure S9 SDS-PAGE Confirmation of Purified mCXCL10-TrxA SUMO Fusion Protein 16

Figure S10 Flow Cytometry Gating Scheme for CD4^+^ and CD8^+^ T-cells 17

Figure S11 De-Polymerization of Hydrogels Equipped with Di-azide Ester Crosslinkers 18

*Literature Cited* 19

# General Synthetic Information

Chemical reagents and solvents were purchased from either Sigma-Aldrich or Fisher Scientific and used as received unless otherwise noted. Peptide synthesis reagents were purchased from either ChemPep or Chem-Impex and used as received. Deionized water (dH_2_O) was generated by a U.S. Filter Corporation Reverse Osmosis System with a Desal membrane. Synthetic chemical reactions were performed under a nitrogen atmosphere in oven-dried glassware and stirred with a Teflon-coated magnetic stir bar unless otherwise noted. Solvents were removed *in vacuo* with a Büchi Rotovapor R-3 equipped with a V-700 vacuum pump and V-855 vacuum controller and a Welch 1400 DuoSeal Belt-Drive high vacuum pump. Microwave-assisted peptide synthesis was performed on a CEM Liberty 1. Semi-preparative reversed-phase high-pressure liquid chromatography (RP-HPLC) was performed on a Dionex Ultimate 3000 equipped with a variable multiple wavelength detector, automated fraction collector, and Thermo 5 µm Synchronis silica 250 x 21.2 mm C18 column. Lyophilization was performed on a LABCONCO FreeZone 2.5 Plus freeze-dryer equipped with a LABCONCO rotary vane 117 vacuum pump. Matrix-assisted laser desorption/ionization time of flight (MALDI-TOF) mass spectrometry was performed in reflectron positive ion mode on a Bruker AutoFlex II using a matrix of α-cyano-4-hydroxycinnamic acid:2,5-dihydroxy benzoic acid (2:1). Fluorescence readings were acquired on a SpectraMax M5 spectrometer using Thermo Scientific Nunc black polypropylene 96-well plates. Protein expression was performed in a Thermo Scientific MaxQ 4000 shaker incubator. Bacterial cells were sonicated using a Qsonica Q500 Sonicator with a 1/4" Microtip Probe.

# Synthesis of Previously Reported Hydrogel Compounds Used in this Work


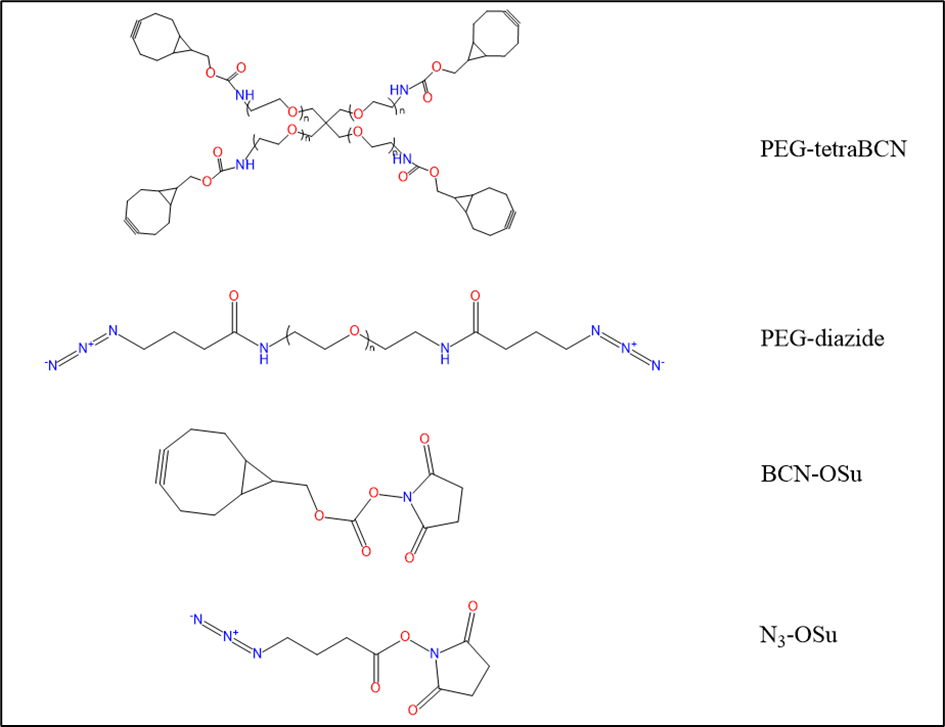


SPAAC-functionalized hydrogel components [Poly(ethylene glycol) tetrabicyclononyne (PEG-tetraBCN, M_n_ ~ 20,000 Da), poly(ethylene glycol) diazide (PEG-diazide, Mn ~ 3,400 Da), (1R,8S,9s)-bicyclo[6.1.0]non-4-yn-9-ylmethyl (2,5-dioxopyrrolidin-1-yl) carbonate (BCN-OSu), and 2,5-dioxopyrrolidin-1-yl 4-azidobutanoate (N_3_-OSu)] were synthesized as previously reported.^1-5^

# Fmoc Solid-Phase Peptide Synthesis

For the instances when Fmoc-GGGGRS-NH_2_ was synthesized in-house, a CEM Liberty 1 was used to perform microwave-assisted Fmoc solid phase peptide synthesis (SPPS, 1 mmol scale) to generate the polypeptide. Fmoc deprotection was performed in 20% piperidine (v/v) in dimethylformamide (DMF) with 1-hydroxybenzotriazole (HObT, 0.1 M, 90 °C, 90 sec). Amino acids were coupled to resin-bound peptides upon treatment (75 °C, 5 min) with Fmoc-protected amino acid (2 mmol, 4x), 2-(1H-benzotriazol-1-yl)-1,1,3,3-tetramethyluronium hexaﬂuorophosphate (HBTU, 2 mmol, 4x), and *N*,*N*-diisopropylethylamine (DIEA, 2 mmol, 4x) in a mixture of DMF (9 mL) and *N*-Methyl-2-pyrrolidone (NMP, 2 mL).

# Method S2 Synthesis of Fmoc-GGGGRS-NH_2_


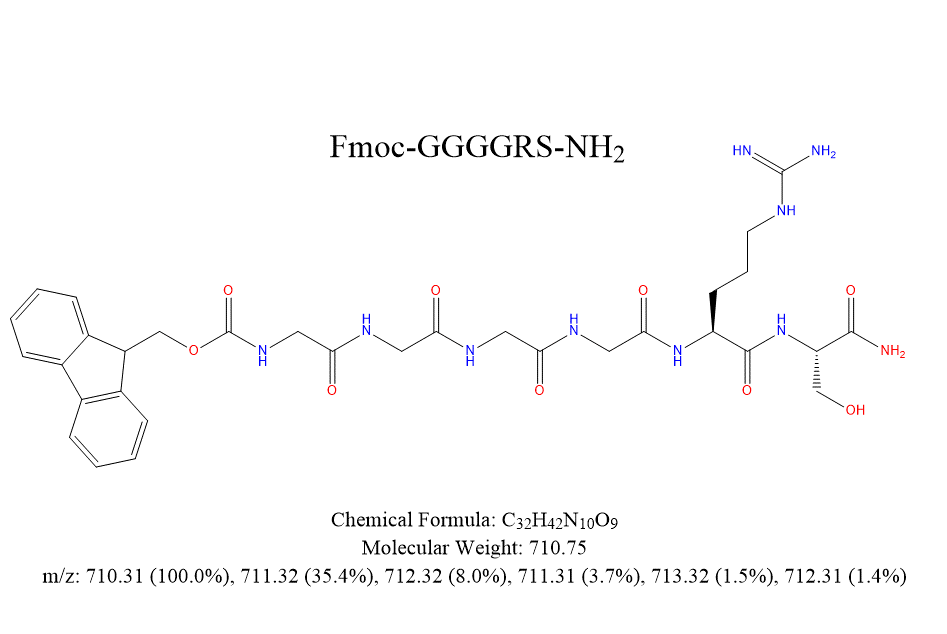


The resin-bound peptide, Fmoc-GGGGRS-NH2, was synthesized in house by Fmoc SPPS (Method S1) on Rink amide resin (1 mmol scale). Reagents input into the Liberty 1 synthesizer were as follows: 6.24g Fmoc-Arg(pbf)-OH (Chempep, 100202) in 18mL DMF, 5.48g Fmoc-Gly-OH (Chempep, 100801) in 35mL DMF and 1.84g Fmoc-Ser(tbu)-OH (Chempep, 101602) in 9mL DMF. 1.35g of Rink amide at 0.74 scale was used. Deprotection solution: 2.162g HObT dissolved in 160mL of 20% Piperidine in DMF. Activator Base: 10.5mL DIEA in 20mL NMP. Activator: 9.48g HBTU dissolved in 50mL DMF. After the synthesis program was completed, the resin was rinsed 3x in DCM. The peptide was cleaved from the resin using 40mL Cleavage Cocktail (95% Trifluoroacetic Acid, 5% H_2_O, 5% Triisopropylsilane) stirring for 2 hours at RT. This cleavage solution was crashed in Di-ethyl Ether (2x), spun down and dried under a nitrogen atmosphere. The crude peptide was purified via RP-HPLC using a 55-minute gradient from 20-100% acetonitrile:H2O; lyophilization yielded the final product (Fmoc-GGGGRS-NH2) as a white solid. Peptide purity was confirmed using MALDI-TOF. Expected MW: 710.31 Da. Actual: 711.30 Da


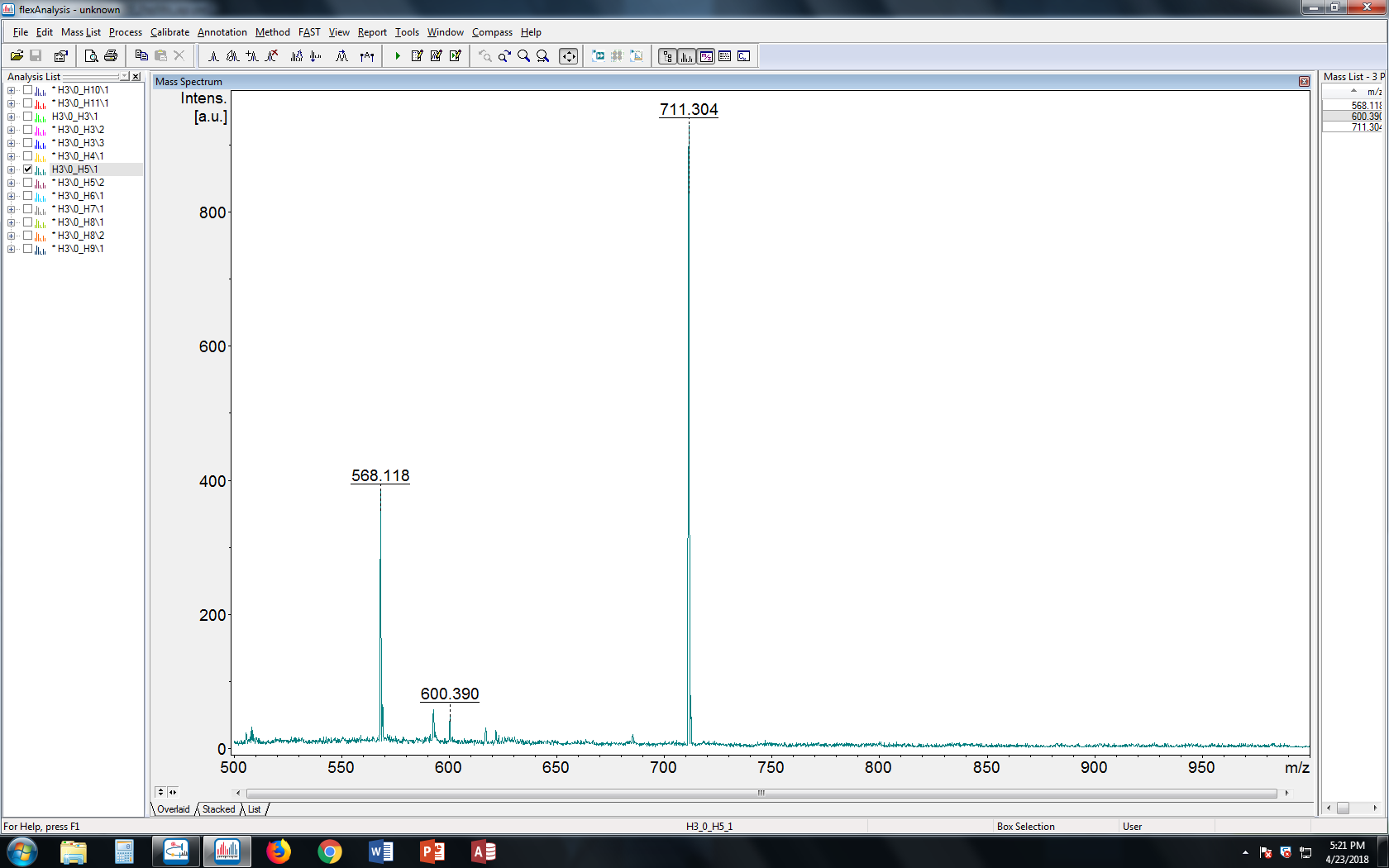


MW: 711.30

# Method S3 Synthesis of DEAC-OH


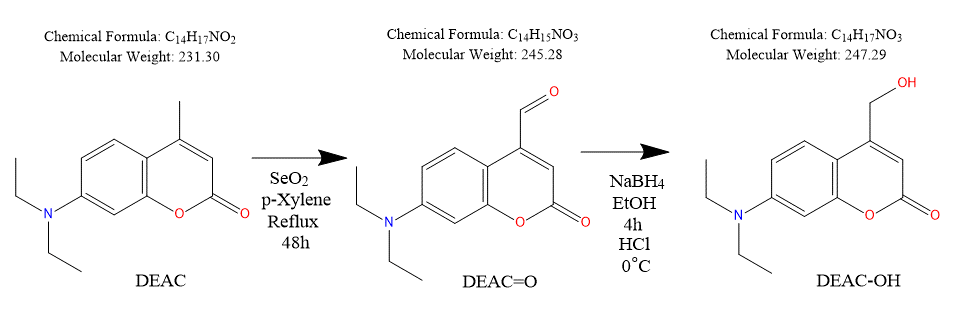


Simplified synthesis scheme of 7-Diethylamino-4-(hydroxymethyl) coumarin (DEAC-OH, MW: 247.29 Da) synthesized in-house utilizing 7-Diethylamino-4-methylcoumarin (DEAC, 231.29 Da) (Fisher Sci, 50534181) as starting material. Briefly, DEAC (0.5 g; 2.16 mmol) was combined with selenium dioxide (0.7 g; 6.3 mmol; 3 eq) in a round bottom flask and dissolved in p-Xylene (30 mL). The solution was refluxed for 48 hours under vigorous stirring and quickly became dark brown over the course of 3 hours. After 48 hours, the mixture was filtered and concentrated under reduced pressure. The residual brown oil was dissolved in Ethanol and Sodium Borohydride (0.985 g; 26 mmol) was added. The solution was stirred for 4 hours before careful hydrolysis with HCl (1 M; 2.5 mL) at 0 °C. The solution was then diluted into water and extracted with 3 portions of dichloromethane before drying with MgSO_4_, filtration, and concentration under reduced pressure. The result was purified using a 0-70% gradient of ethyl acetate in hexanes to provide the product, DEAC-OH (290 mg; 54% yield).

# Figure S1 Intracortical Hydrogel Injections in Mouse Brains *in vivo*

#
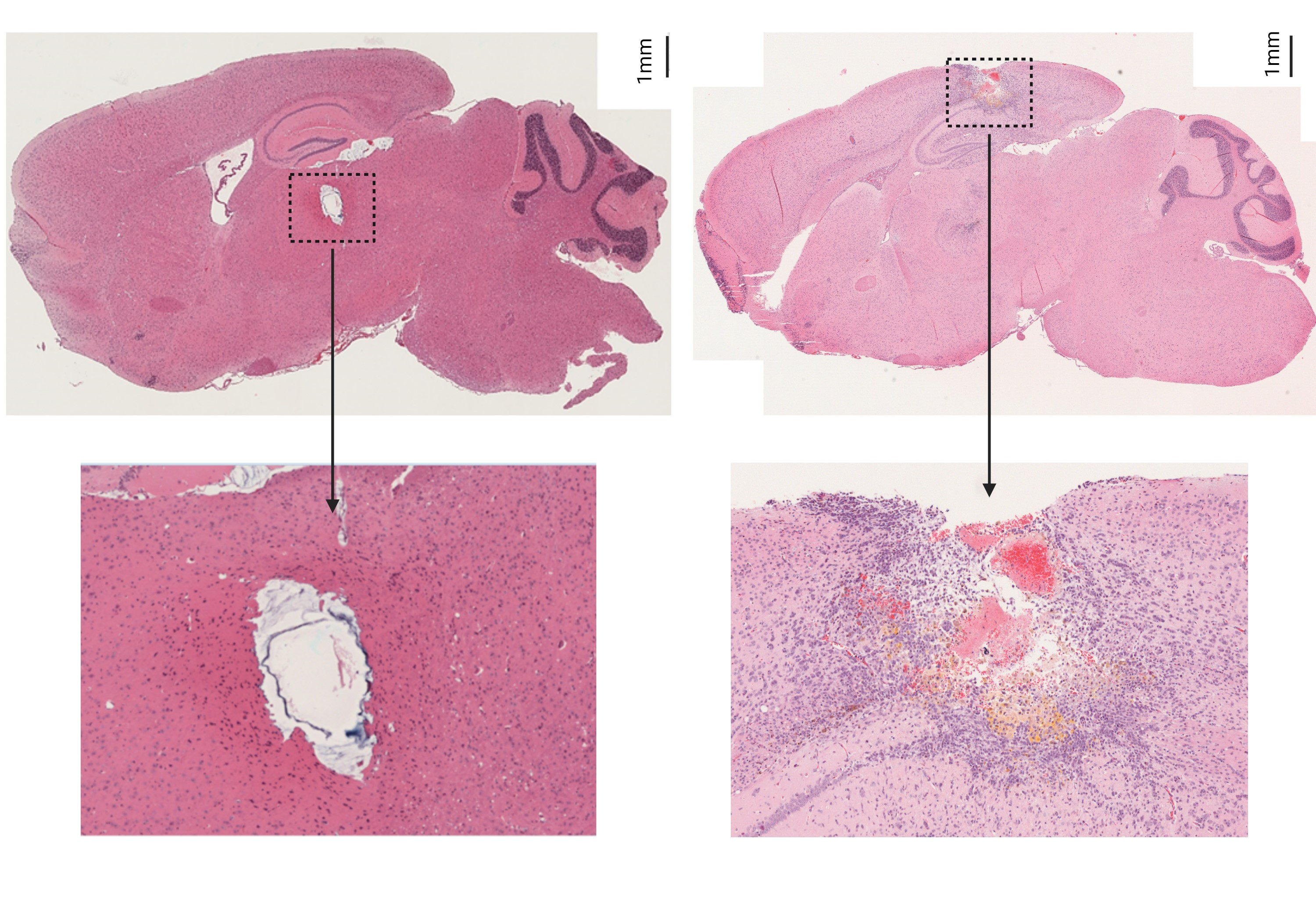


In total, we conducted over a dozen liquid hydrogel injections into living murine brains at different depths to demonstrate the ease and reproducibility of initiating *in situ* SPAAC hydrogel polymerization within a physically constrained space. Gelation occurred optimally when the solution reached the mouse's body temperature of 37°C. In the representative images above, including those shown in **Figure 1**, our hydrogels were positioned at various depths within the murine cortex by adjusting the stopper on the Hamilton syringe. The residual gels were identifiable through microscopy within the injection cavity, and no adverse effects were observed before euthanasia.

# Figure S2 Anti-CD47 Antibody Blockade Results in Phagocytosis of Human pHGG Xenograft Cells by Murine Macrophages


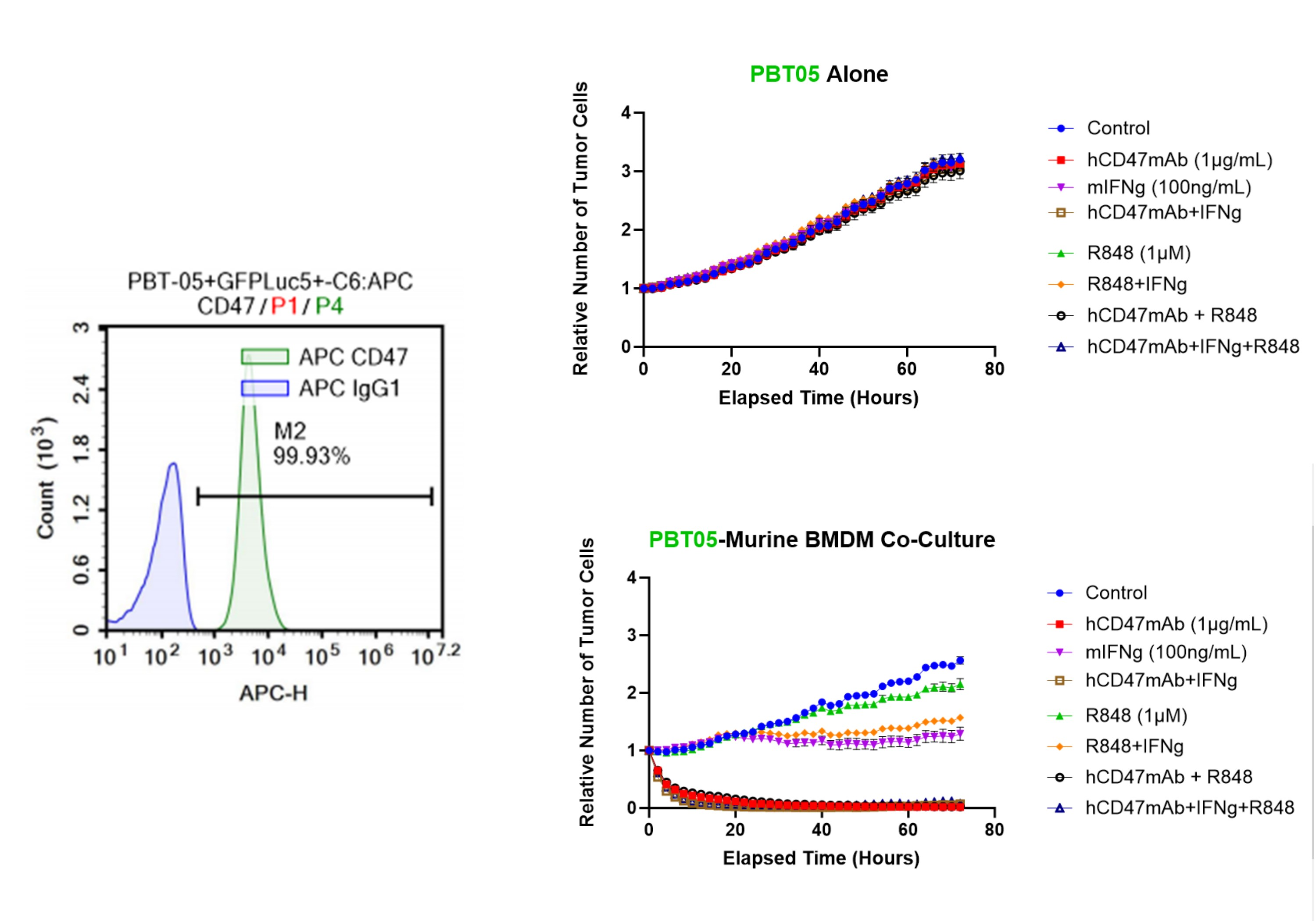


#
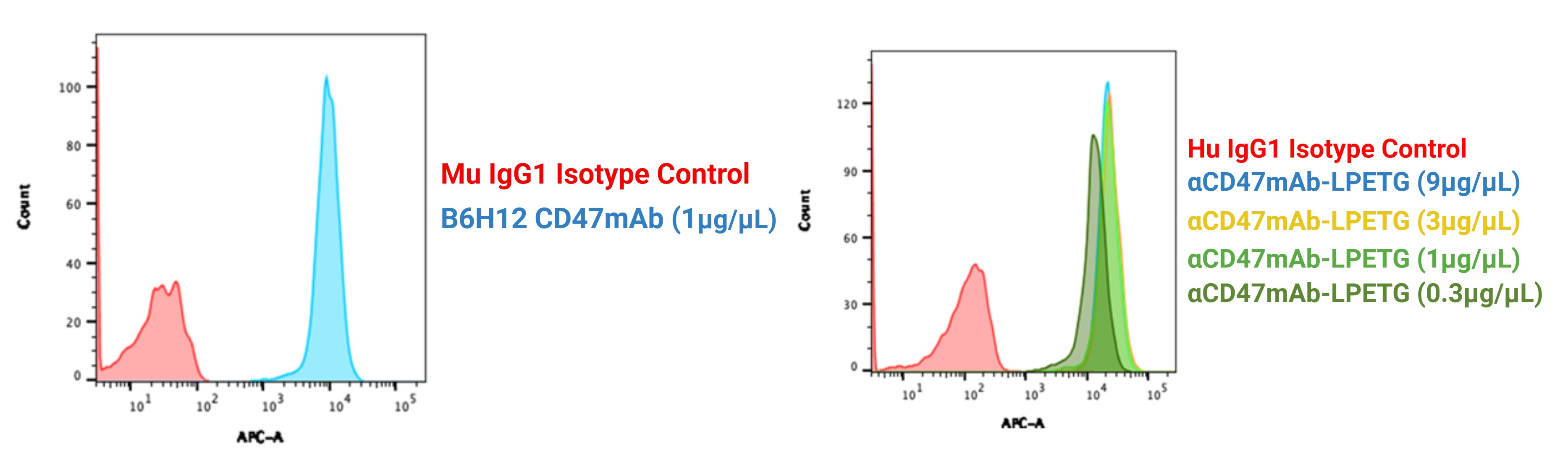


(Top Left) Flow cytometry confirms CD47 expression on the pediatric high-grade glioma (pHGG) xenograft line, PBT-05. (Top Right) GFP^+^ PBT-05 cells were cultured alone or in 1:1 ratio co-cultures with M0/M2 bone marrow-derived macrophages (BMDM), treated with various immunomodulators to optimize conditions for inducing phagocytosis of PBT-05, with CD47mAb B6H12 clone (BioXcell) showing the best single-agent efficacy without interferon-gamma (IFNg) polarization.^6-10^ (Bottom Left) Confirmation of the binding ability of the APC-labeled CD47mAb B6H12 clone (eBiosciences) on PBT-05. (Bottom Right) Validation of the binding ability of the in-house-produced recombinant αCD47-LPETG mAb (2.3 D11 clone) at various dilutions.

# Figure S3 IgG-AF594 Diffusion from 5.2-8% (w/v) PEG-tBCN Hydrogels

#
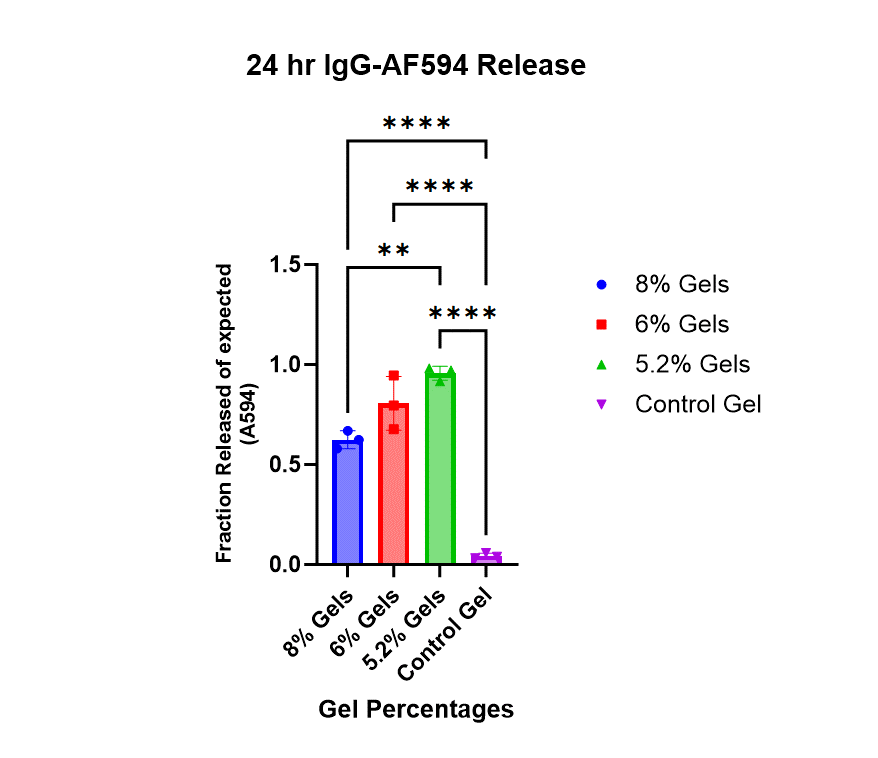


This graph depicts the release fraction of IgG-AlexaFluor 594 (~160kDa) from PEG-tBCN hydrogels formulated at 5.2-8% (w/v) over 24 hours. The gels were cast in 25µL volumes, allowed to settle for 1 hour at room temperature, and then immersed in 50µL of PBS release media overnight. The released protein fraction was quantified using linear regression with an IgG-AlexaFluor 594 standard in PBS, and the 594nm signal was used for protein quantification. An 8% (w/v) untreated control gel was included to account for false fluorescent signals. Data are presented as mean ± SD for n=3 replicates, and statistical significance was determined by ordinary one-way ANOVA followed by Holm-Šídák posthoc correction, with p-values less than 0.01 (** through ****) displayed.

# Figure S4 Expression of “αCD47mAb-LPETG” IgG1 with C-terminal Sortase Motifs


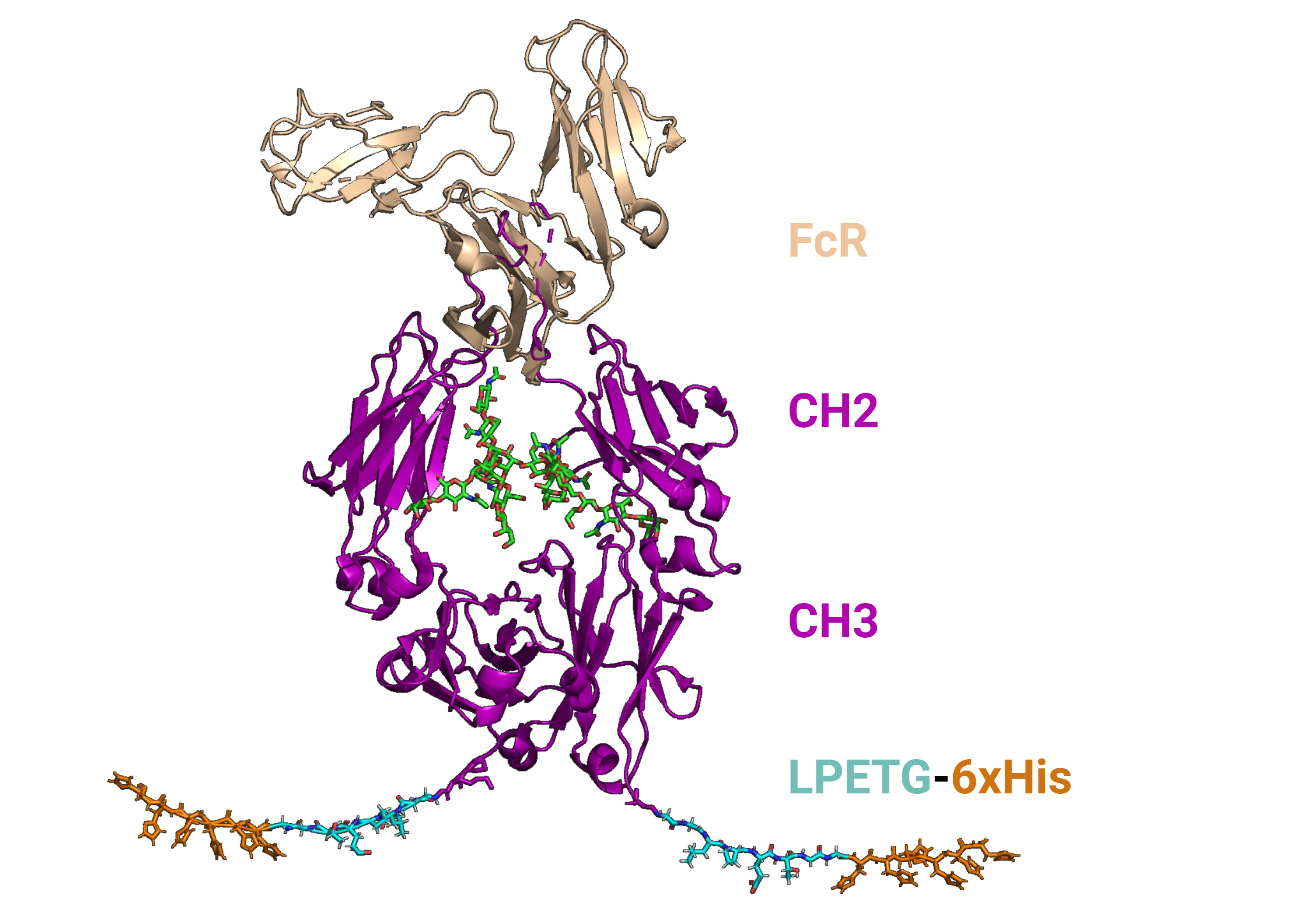

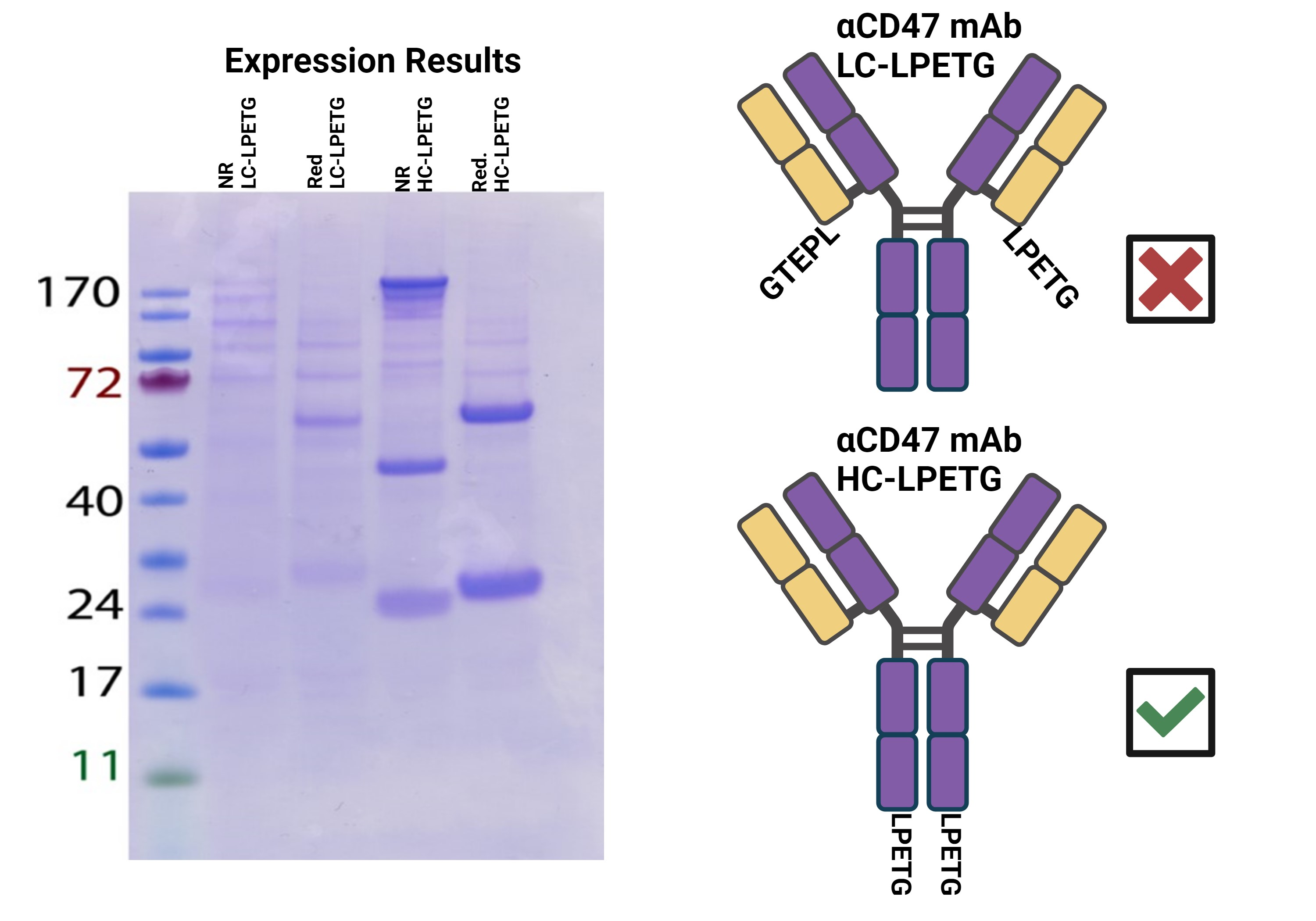


(Left) This figure illustrates the expression of two variants of the CD47mAb 2.3D11 clone, “αCD47-LPETG”, developed for our study. We showcase successful expression of the antibody with LPETG-6xHis modifications on the C-termini of the heavy chains (columns 3 and 4, non-reduced and reduced, respectively) but unsuccessful expression of the variant with an LPETG motif encoded at the C-termini of the light chains (columns 1 and 2, non-reduced and reduced, respectively). The structural illustration on the right depicts the interactions of the IgG1 Fc domain with the FcγR at its CH2 domain. This interaction occurs distal to the LPETG-6xHis modifications at the very C-terminus of the heavy chains.^11-13^

# Figure S5 MALDI-TOF Confirmation of PolyG-3,4azidoester Synthesis

**Expected Actual**

**
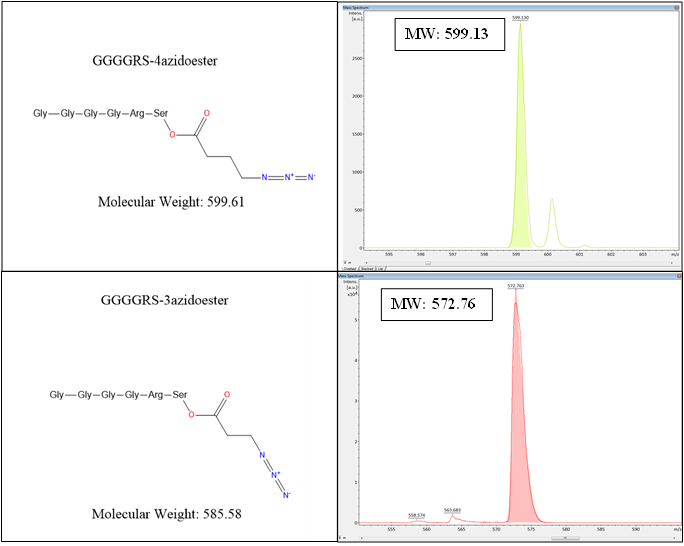
**

**
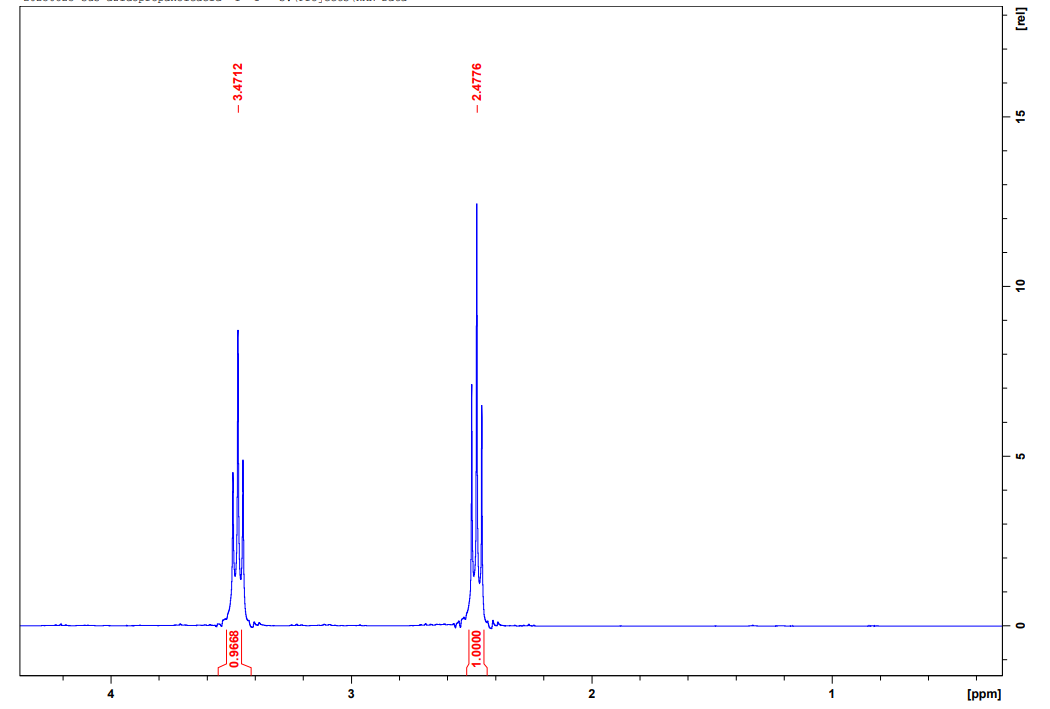
**

(Top) MALDI-TOF mass spectrum traces were obtained for each of the synthesized products. PolyG-4azidoester was found to match its expected molecular weight (MW: 599.13 Da), and the resulting lyophilized product appeared as a light-yellow oil, as anticipated. However, PolyG-3azidoester appeared to be missing approximately 14 Da, making its molecular weight closer to that of PolyG-2azidoester (MW: 571.56 Da) rather than its own expected molecular weight (MW: 585.58 Da). (Bottom) H-NMR on our stock of 3-azidopropanic (Synthonix, A1939) demonstrates the presence of two triplets at 3.47ppm and 2.47ppm, confirming the identity of 3-azidopropanic acid. We conclude the observed mass discrepancy may be an artifact of the spectroscopy process.

# Figure S6 Amino Acid Sequence of Human CCL2 STEPL Fusion Protein

*hCCL2-LPETG-Sa-SrtA-6xHis*:

QPDAINAPVTCCYNFTNRKISVQRLASYRRITSSKCPKEAVIFKTIVAKEICADPKQKWVQDSMDHLDKQTQTPKTLELPETGGGSGGSGGSGGSGGSQAKPQIPKDKSKVAGYIEIPDADIKEPVYPGPATPEQLNRGVSFAEENESLDDQNISIAGHTFIDRPNYQFTNLKAAKKGSMVYFKVGNETRKYKMTSIRDVKPTDVEVLDEQKGKDKQLTLITCDDYNEKTGVWEKRKIFVATEVKHHHHHH

The complete amino acid sequence of mature human CCL2 (aa 24-99, derived from Peprotech 300-04) fused with sortase in the pSTEPL expression plasmid.^14,15^ The sequence begins with CCL2 in red, separated from the rest of the fusion protein by a GGS_5_ linker (black). The LPETG sortase recognition motif is labeled in blue, a truncated sortase enzyme is labeled in green, and a C-terminal 6x-His tag is labeled in orange. It should be noted that bacterial methionine aminopeptidase (MAP) may not always remove the formyl-methionine used to initiate translation of this protein.^16^ Although this is not a problem for every protein, chemokine-receptor signaling is dependent upon the preservation of the native N-terminus (i.e.: H-Gln).^17^ We did not perform receptor-mediated assays with CCL2, but addressing this was necessary for our studies utilizing CXCL10 in **Figure S8**.

# Figure S7 Conjugating PolyG-4azidoester to Human CCL2 via STEPL


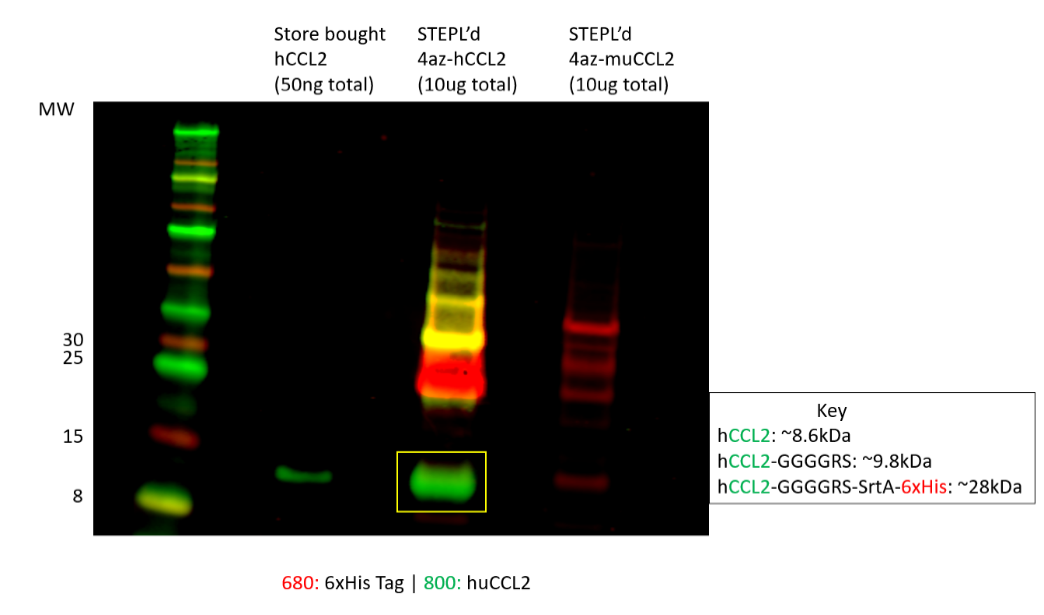


A Western blot was performed to confirm the expression and modification of the human CCL2 pSTEPL fusion protein with PolyG-4azidoester attachments.^14,15^ The blot consisted of three lanes: a human CCL2 positive control (Thermofisher, RP-8648), the collected flow-through from an on-column reaction between human CCL2-STEPL fusion with PolyG-4azidoester, and a collected flow-through from an on-column reaction between murine CCL2-STEPL fusion (sequence derived from Peprotech, 250-10) with PolyG-4azidoester as a negative control. The left lane showed the positive control detected by a polyclonal human CCL2 antibody (RnD Systems, AF-279-SP) in the green channel. The middle column showed the human CCL2 separated from the greater STEPL fusion protein and visible in the green channel (highlighted in a yellow box). The right column showed the negative control, with murine CCL2 labeled non-specifically with the 6x-His Tag antibody (Abcam, ab18184) in the red channel but not detected by the human-specific CCL2 antibody.

# Figure S8 Amino Acid Sequence, Model and Processing of mCXCL10 SUMO Fusion

*6xHis-TrxA- SUMO-mCXCL10-LPETG-Strep*:

HHHHHHGSDKIIHLTDDSFDTDVLKADGAILVDFWAEWCGPCKMIAPILDEIADEYQGKLTVAKLNIDQNPGTAPKYGIRGIPTLLLFKNGEVAATKVGALSKGQLKEFLDANLAGTSDSEVNQEAKPEVKPEVKPETHINLKVSDGSSEIFFKIKKTTPLRRLMEAFAKRQGKEMDSLRFLYDGIRIQADQTPEDLDMEDNDIIETHREQIGGIPLARTVRCNCIHIDDGPVRMRAIGKLEIIPASLSCPRVEIIATMKKNDEQRCLNPESKTIKNLMKAFSQKRSKRAPGGSGGSLPETGWSHPQFEK


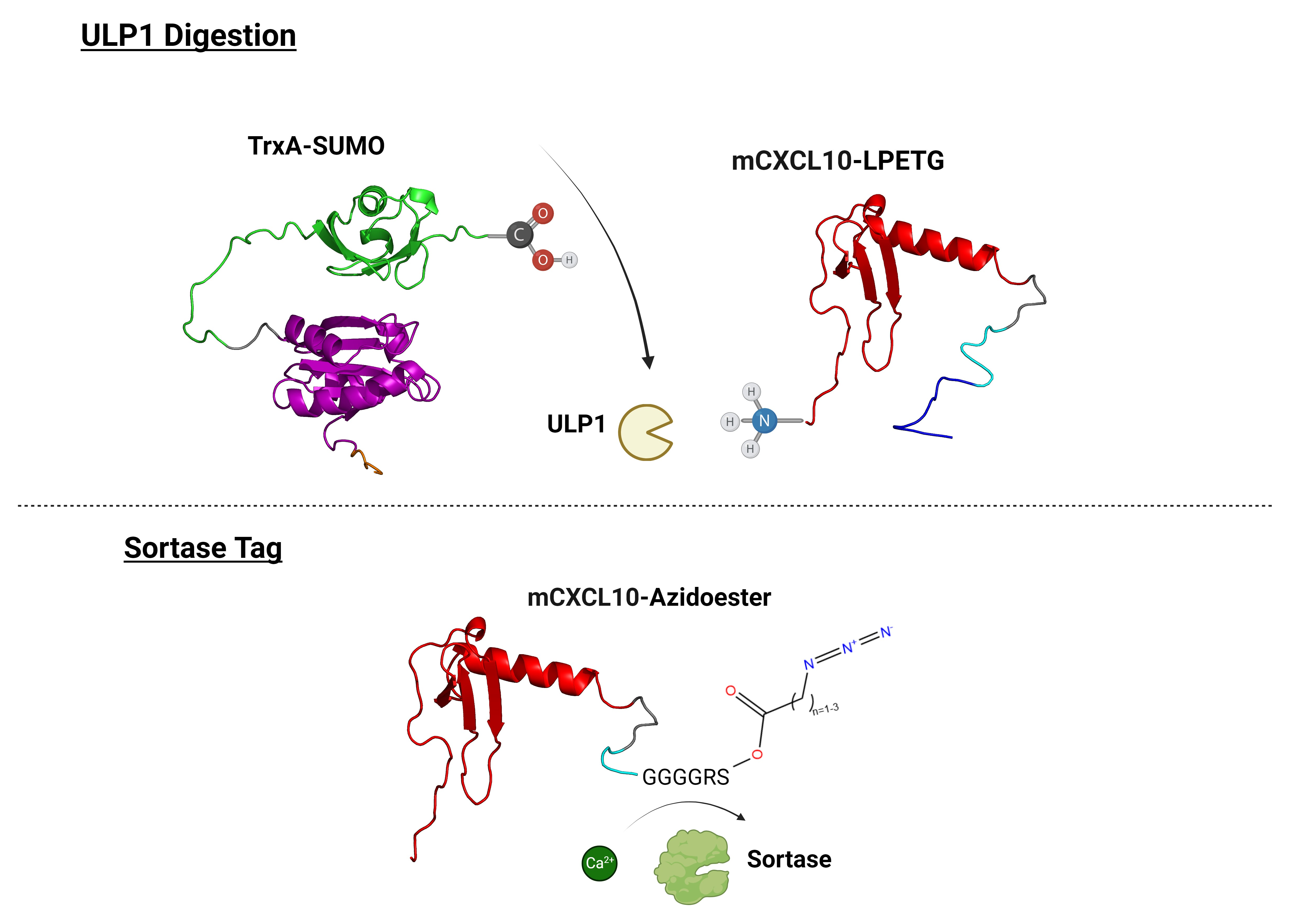

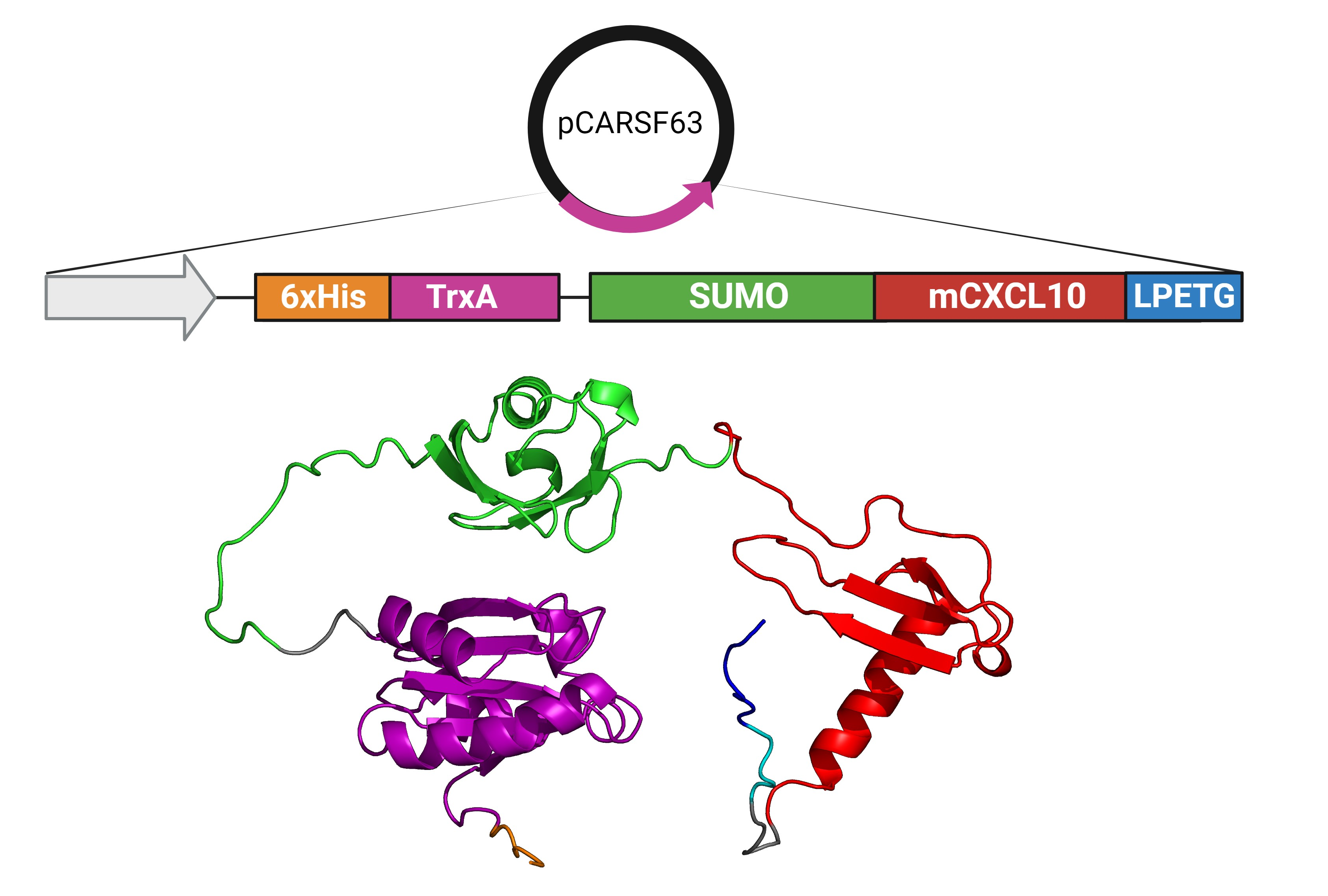


(Top, Bottom Left) The complete amino acid sequence of mature, murine CXCL10 (aa 22-98, derived from Peprotech 250-16) fused with Thioredoxin and SUMO within the pCARSF63 expression plasmid is shown.^18-20^ The fusion protein begins with an N-terminal 6x-His tag in orange, followed by Thioredoxin in purple, and then SUMO in Green. (Bottom Right) The terminal “GG” of SUMO are subject to specific cleavage by SUMO protease (ULP1), releasing what immediately follows with a native N-terminus (i.e., H-Ile).^18,20^ The sequence of murine CXCL10 is labeled in red, followed by LPETG in light blue and a Strep Tag in navy blue. Upon overnight digestion of this fusion protein by ULP1 (Thermofisher, 12588018)^21^, the resulting chemokine has a native N-terminus which preserves receptor signaling and biological activity.^17^ We then performed traditional sortase tagging to add a PolyG-azidoester to its C-terminus.^15^

# Figure S9 SDS-PAGE Confirmation of Purified mCXCL10-TrxA SUMO Fusion Protein

**
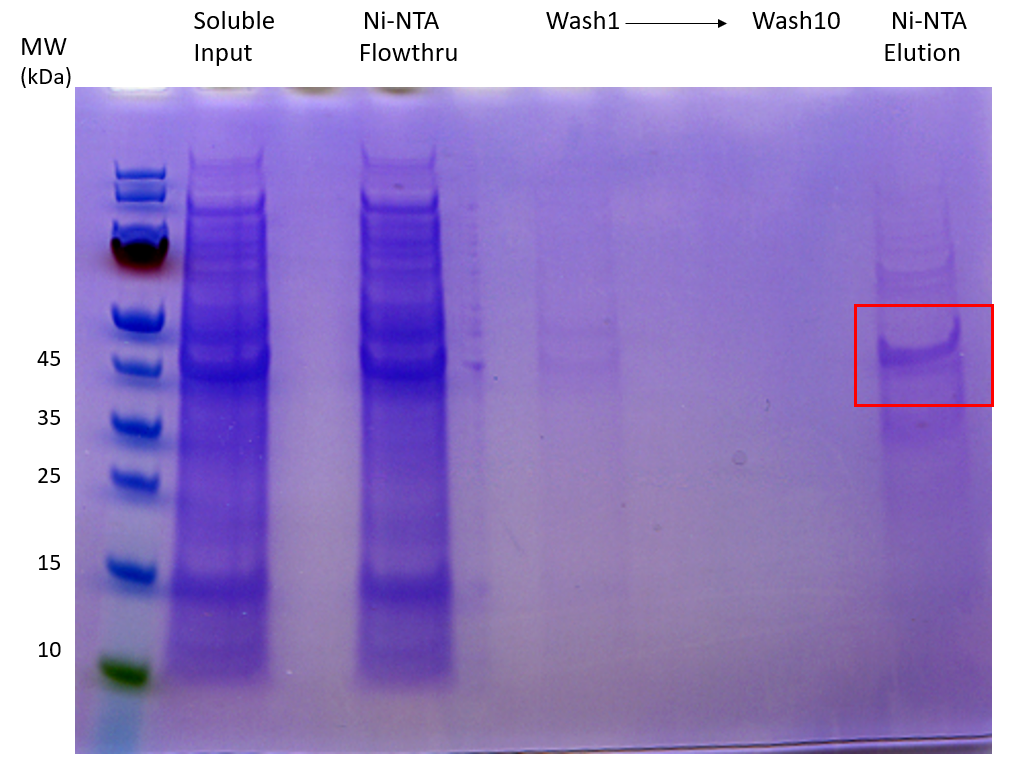
**

This SDS-PAGE gel depicts the purification process after the expression of the SUMO fusion protein in Shuffle T7 Express *E. coli* (NEB). The purification involves a Ni-NTA pulldown and repeated wash steps. The purified fusion protein is collected in the Ni-NTA elution lane on the far right, which is highlighted in a red box. The purified protein will then be stored for future ULP1 cleavage and sortagging.

# Figure S10 Flow Cytometry Gating Scheme for CD4^+^ and CD8^+^ T-cells

**
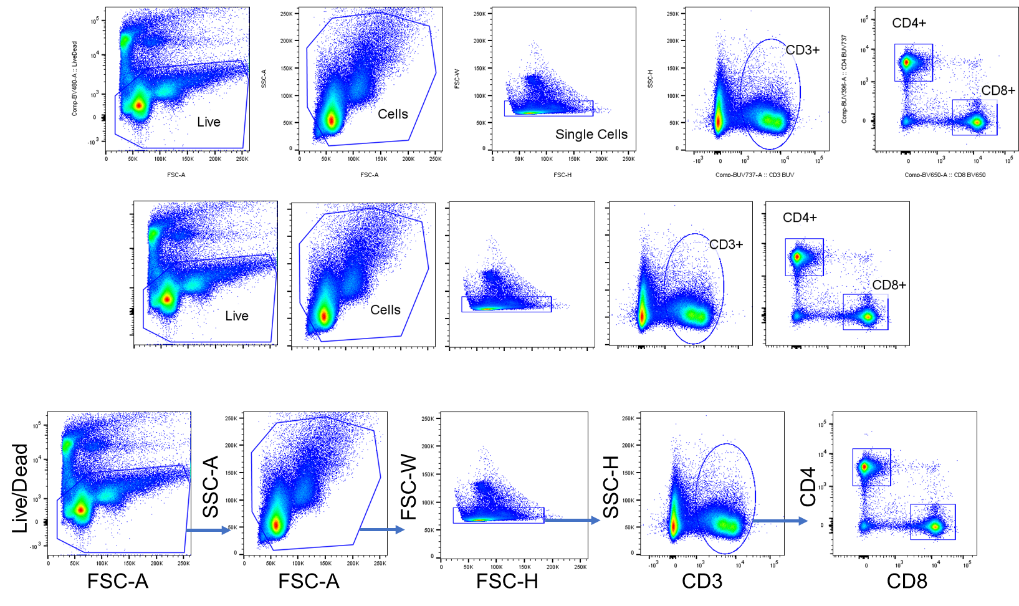
**

The image depicts the gating scheme used to identify T-cell populations in B16 melanoma flank tumors at the conclusion of our *in vivo* study. All antibodies and viability dyes were purchased from Biolegend: Zombie Aqua^TM^ live dead, CD8a (53-6.7), CD4 (RM4-5), and CD3 (17A2). 5 x10^6^ cells were stained for surface or intracellular proteins by incubating cells with antibodies diluted in PBS + 2% BSA for 45 minutes on ice. Cells were then washed 3x in flow cytometry stain buffer and fixed with 2% PFA for 20 minutes prior to acquisition on a LSRII Fortessa (BD Biosciences). Samples were analyzed with FlowJo V10 software.

# Figure S11 De-Polymerization of Hydrogels Equipped with Di-azide Ester Crosslinkers


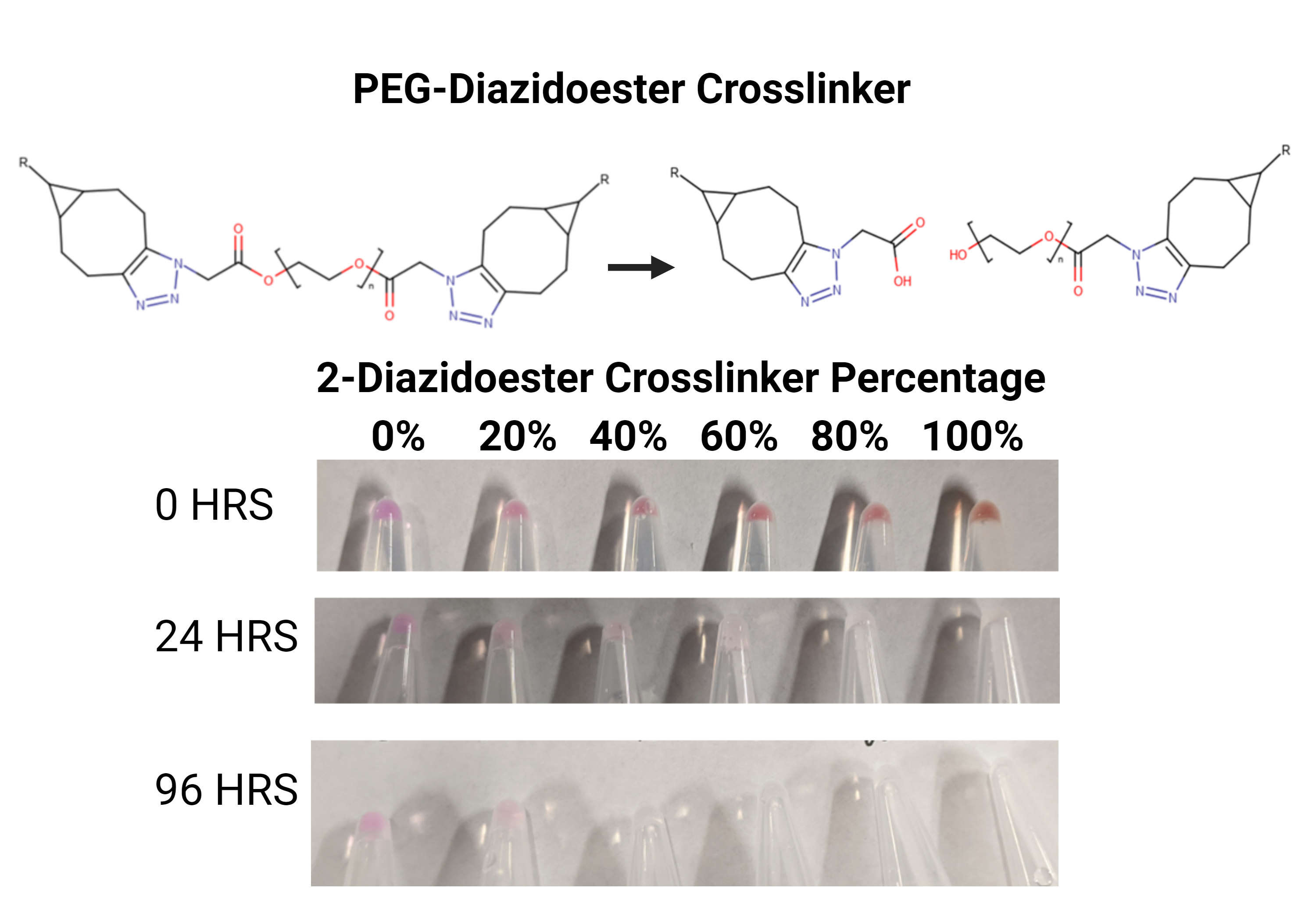


Top: This schematic illustrates the hydrolysis process of our proposed PEG-Diazidoester crosslinker, which serves to connect the 4-arm PEG network. The synthesis of these crosslinkers can be achieved using either Mono PEG-OH or Di PEG-OH, followed by functionalization with azidoesters through carbodiimide chemistry.^22^ Bottom: We showcase the depolymerization of fluorescently labeled PEG-tBCN hydrogels over time, correlating it with elapsed time *and* the ratio of PEG-Diazidoester to the original amidated PEG-Diazide.^23^

# *Literature Cited*

1. DeForest CA, Tirrell DA. A photoreversible protein-patterning approach for guiding stem cell fate in three-dimensional gels. *Nat Mater*. May 2015;14(5):523-31. doi:10.1038/nmat4219

2. Badeau BA, Comerford MP, Arakawa CK, Shadish JA, DeForest CA. Engineered modular biomaterial logic gates for environmentally triggered therapeutic delivery. *Nat Chem*. Mar 2018;10(3):251-258. doi:10.1038/nchem.2917

3. Shadish JA, Strange AC, DeForest CA. Genetically Encoded Photocleavable Linkers for Patterned Protein Release from Biomaterials. *J Am Chem Soc*. Oct 2 2019;141(39):15619-15625. doi:10.1021/jacs.9b07239

4. Agard NJ, Prescher JA, Bertozzi CR. A strain-promoted [3 + 2] azide-alkyne cycloaddition for covalent modification of biomolecules in living systems. *J Am Chem Soc*. Nov 24 2004;126(46):15046-7. doi:10.1021/ja044996f

5. Dommerholt J, Rutjes F, van Delft FL. Strain-Promoted 1,3-Dipolar Cycloaddition of Cycloalkynes and Organic Azides. *Top Curr Chem (Cham)*. Apr 2016;374(2):16. doi:10.1007/s41061-016-0016-4

6. Liu J, Wang L, Zhao F, et al. Pre-Clinical Development of a Humanized Anti-CD47 Antibody with Anti-Cancer Therapeutic Potential. *PLoS One*. 2015;10(9):e0137345. doi:10.1371/journal.pone.0137345

7. Hutter G, Theruvath J, Graef CM, et al. Microglia are effector cells of CD47-SIRPalpha antiphagocytic axis disruption against glioblastoma. *Proc Natl Acad Sci U S A*. Jan 15 2019;116(3):997-1006. doi:10.1073/pnas.1721434116

8. Chao MP, Takimoto CH, Feng DD, et al. Therapeutic Targeting of the Macrophage Immune Checkpoint CD47 in Myeloid Malignancies. *Front Oncol*. 2019;9:1380. doi:10.3389/fonc.2019.01380

9. Maute R, Xu J, Weissman IL. CD47-SIRPalpha-targeted therapeutics: status and prospects. *Immunooncol Technol*. Mar 2022;13:100070. doi:10.1016/j.iotech.2022.100070

10. Nishiga Y, Drainas AP, Baron M, et al. Radiotherapy in combination with CD47 blockade elicits a macrophage-mediated abscopal effect. *Nat Cancer*. Nov 2022;3(11):1351-1366. doi:10.1038/s43018-022-00456-0

11. Kiyoshi M, Caaveiro JM, Kawai T, et al. Structural basis for binding of human IgG1 to its high-affinity human receptor FcgammaRI. *Nat Commun*. Apr 30 2015;6:6866. doi:10.1038/ncomms7866

12. Caaveiro JMM, Kiyoshi, M., Tsumoto, K. High-resolution crystal structure of Fc bound to its human receptor Fc-gamma-RI.

13. Osorio JC, Smith P, Knorr DA, Ravetch JV. The antitumor activities of anti-CD47 antibodies require Fc-FcgammaR interactions. *Cancer Cell*. Dec 11 2023;41(12):2051-2065 e6. doi:10.1016/j.ccell.2023.10.007

14. Warden-Rothman R, Caturegli I, Popik V, Tsourkas A. Sortase-tag expressed protein ligation: combining protein purification and site-specific bioconjugation into a single step. *Anal Chem*. Nov 19 2013;85(22):11090-7. doi:10.1021/ac402871k

15. Popp MW, Antos JM, Ploegh HL. Site-specific protein labeling via sortase-mediated transpeptidation. *Curr Protoc Protein Sci*. Apr 2009;Chapter 15:15 3 1-15 3 9. doi:10.1002/0471140864.ps1503s56

16. Wingfield PT. N-Terminal Methionine Processing. *Curr Protoc Protein Sci*. Apr 3 2017;88:6 14 1-6 14 3. doi:10.1002/cpps.29

17. Kleist AB, Getschman AE, Ziarek JJ, et al. New paradigms in chemokine receptor signal transduction: Moving beyond the two-site model. *Biochem Pharmacol*. Aug 15 2016;114:53-68. doi:10.1016/j.bcp.2016.04.007

18. Lu Q, Burns MC, McDevitt PJ, et al. Optimized procedures for producing biologically active chemokines. *Protein Expr Purif*. Jun 2009;65(2):251-60. doi:10.1016/j.pep.2009.01.017

19. Mirdita M, Schutze K, Moriwaki Y, Heo L, Ovchinnikov S, Steinegger M. ColabFold: making protein folding accessible to all. *Nat Methods*. Jun 2022;19(6):679-682. doi:10.1038/s41592-022-01488-1

20. Guerreo Fea. Tandem SUMO fusion vectors for improving soluble protein expression and purification *Protein Expr Purif*. 2015;116:42-49.

21. Consortium TU. UniProt: the Universal Protein Knowledgebase in 2023. *Nucleic Acids Research*. 2022;51(D1):D523-D531. doi:10.1093/nar/gkac1052

22. Neises B, Steglich W. Simple Method for the Esterification of Carboxylic Acids. *Angewandte Chemie International Edition in English*. 1978;17(7):522-524. doi:10.1002/anie.197805221

23. Schoenmakers RG, van de Wetering P, Elbert DL, Hubbell JA. The effect of the linker on the hydrolysis rate of drug-linked ester bonds. *J Control Release*. Mar 5 2004;95(2):291-300. doi:10.1016/j.jconrel.2003.12.009
